# Adsorbent Properties of Porous Boron Nitride and Activated Carbon: A Comparative Study

**DOI:** 10.1021/acsomega.4c02625

**Published:** 2024-10-11

**Authors:** Christian Bläker, Tim Jähnichen, Jan Hojak, Laura Gehrke, Christoph Pasel, Thomas Paschke, Frieder Dreisbach, Dirk Enke, Dieter Bathen

**Affiliations:** † Chair of Thermal Process Engineering, University of Duisburg-Essen, Duisburg 47057, Germany; ‡ Institute of Chemical Technology, Leipzig University, Leipzig 04103, Germany; § TA Instruments - Waters GmbH, Hüllhorst 32609, Germany

## Abstract

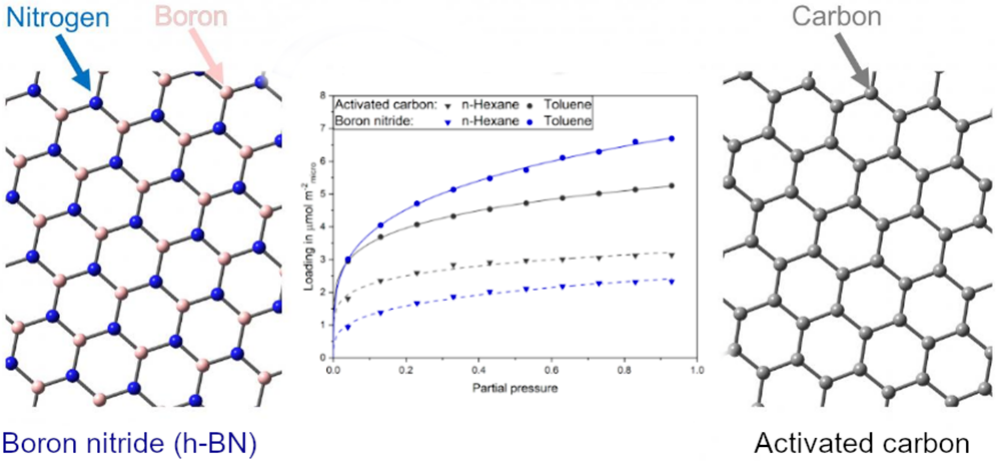

Porous boron nitrides possess beneficial properties such as high thermal and chemical stability which are critical for applications in adsorption processes. In order to assess possible fields of applications, trace-level adsorption isotherms of different hydrocarbons on two synthesized porous boron nitrides and two commercial activated carbons are compared. By normalizing the adsorptive loadings on the micropore surface area, superior adsorption performances of the BN materials on polar and aromatic adsorptives with up to 50% higher loadings compared to the activated carbons can be shown. Nonpolar adsorptives, on the other hand, feature higher specific loadings on the activated carbon. Consequently, the size of the micropore surface appears to be decisive for nonpolar adsorptives, while the higher polarity of the boron nitrides is the dominant influencing factor for the adsorption of polar and aromatic components. For an energetic study of the adsorbents, calorimetric experiments were performed to identify and discuss adsorbent-adsorptive interactions. While the initial heat of adsorption of the nonpolar *n*-hexane is lower on the boron nitride than on the activated carbon due to a less favorable spatial arrangement, toluene shows comparable values on both adsorbent classes and the polar acetone shows higher values on the polar boron nitride. Considering technical applications in adsorption technology, the thermal stability of the boron nitrides is investigated using spontaneous ignition temperatures and points of initial oxidation. Here, the porous boron nitrides with oxidation temperatures above 900 °C show about 400 °C higher values and thus a significantly higher thermal stability.

## 1 Introduction

In addition to the modification of well-known adsorbents such as activated carbons,^1^ zeolites,^2^ and silica-gels, new materials such as metal organic frameworks (MOFs),^3^ covalent organic frameworks (COFs), and porous boron nitrides (BN)^4^ show promising solutions for complex applications in adsorption technology.^5,6^ Porous BN has the potential to be produced cost-efficient in large quantities.^7^ The material shows advantageous properties such as high chemical and thermal stability, as well as high specific surface areas.^8−11^ Compared to activated carbons, where adsorber bed ignition may occur during adsorption of reactive hydrocarbons, porous BN could show safety benefits due to a higher oxidation stability.

In this study, a comparison between conventional activated carbons and synthesized porous BNs is carried out. The adsorption performance to water and different hydrocarbons up to partial pressures of 1 mbar is investigated. In addition, heat of adsorption and oxidation stability for both materials are compared.

In general, activated carbons and BNs have similar, isostructural modifications whereby the hexagonal modifications graphite and hexagonal BN (h-BN) are predominant at standard conditions. They consist of multiple layers of hexagonal rings stacked on top of each other. For graphite the stacking is AB-stacked^12^ and for h-BN AA’-stacked (ecliptic), whereby an alternation of boron and nitrogen atoms occurs.^13^ In addition to different modifications, h-BN and graphite can be synthesized in multiple dimensions: 0D fullerenes, 1D nanotubes, 2D nanosheets, and 3D formations.^13−17^ Despite similar structures, both materials show different polarities due to the differences in electronegativity in BN, which has an impact on their respective adsorption performance. While graphite is a nonpolar adsorbent, h-BN has polar properties.

In adsorption processes, the porous analogues of graphite and h-BN are more often used since crystalline modifications have very low porosities due to their high structural order.^18^ In the case of graphite, the less-ordered structure activated carbon is well-known, whereas for h-BN an easy synthesis of less-ordered, so-called turbostratic BN (t-BN) was accomplished just recently.^9,19^ However, with decreasing structural order the stability of a material is reduced as well. In a previous work,^20^ we were able to show that highly porous t-BN decomposes in the presence of water and water-vapor. The material reacts with water to boron oxide and ammonia. This reaction leads to a significant reduction in pore volume and BET surface area.^10,21^ To solve this problem, we synthesized a more crystalline, primarily mesoporous t-BN which led to much higher water-vapor stability.^20,22^

In literature, a lot of publications have proposed the use of porous boron nitrides as adsorbents. The focus has mostly been on CO_2_ capture,^23,24^ H_2_ storage,^25,26^ pollutants removal from air^9,27^ and water pollutants removal.^17,28−30^ In most cases, however, only individual isotherms are shown and a comparison with adsorption on typical adsorbents is missing. As an exception, Marchesini et al.^10^ published a comprehensive study on the adsorption of methylcyclohexane (MCH) and other hydrocarbons on three different micromesoporous BNs at 25 °C. By gravimetric analysis the authors were able to show, that adsorption capacity increased with increasing BET surface area (1092 up to 1650 m^2^ g^–1^). The authors suggest that micropores are the most suitable adsorption sites for hydrocarbons in BN. Tian et al.^11^ published gravimetric adsorption isotherms of methane on t-BNs up to pressures of 70 bar. All materials showed similar loadings. Marchesini et al.^8^ investigated high-pressure adsorption of CO_2_. The CO_2_ adsorption capacities of porous BN were significantly lower than those of other adsorbents like MOFs. High-pressure adsorption of hydrogen on porous BN was investigated by Li et al.^31^ The authors assume chemisorptive processes due to a large number of defects within the BN structure, an opened hysteresis, and a generally high hydrogen capacity. In another work, Li et al.^9^ measured the adsorption capacity of benzene at room temperature on porous BNs and compared it with the adsorption on activated carbons. For this purpose, the adsorbents were each placed in a closed vessel containing saturated benzene-vapor while recording their mass changes over time. Porous boron nitride showed a higher total adsorption capacity and was stable over five adsorption–desorption cycles.

Regarding the adsorption performance of nonporous h-BN, some theoretical and practical investigations were carried out. Strange et al.^32^ investigated the adsorption of *n*-alkenes on BN planes. They observed an increase in the heat of adsorption with increasing hydrocarbon chain length. By using dispersion-corrected density functional theory (DFT-D3), Omidirad et al.^33^ showed that defects within the BN planes lead to higher heats of adsorption for polar adsorptives. Chen et al.^34^ investigated the adsorption of aromatic systems on the surface of h-BN with DFT. They showed that the adsorption of aromatic rings is planar to the BN surface. Furthermore, they observed a correlation between the heat of adsorption and the hydrophobicity of BN.

In addition to adsorption properties, oxidation stability of the adsorbent is a major factor for industrial applications. Whereas activated carbons incinerate at temperatures of 300 to 600 °C in oxygen-containing atmospheres, which entails the risk of adsorber bed ignition,^35,36^ porous BNs are temperature stable up to ∼800 °C.^37^ Moreover, BN does not incinerate at those temperatures but instead reacts to thermally stable boron oxide. In order to compare the flammability of activated carbon and porous BN we are using a method for determining the spontaneous ignition temperature (SIT) as described in ASTM D 3466.^38^ At this temperature, a spontaneous oxidation reaction occurs at the materials surface. For different activated carbons SITs of 250 to 550 °C were observed.^39−41^ The method was further developed by Suzin et al.^41^ who additionally defined a point of initial oxidation (PIO). At the PIO, a change in the surface properties of the material can be observed due to oxidation reactions.

In summary, adsorption data on BNs are only sporadically available and their suitability for use in technical processes has hardly been investigated. To investigate potential new application fields of porous BNs and to provide further adsorption data, in this work adsorption measurements of selected polar, nonpolar, and aromatic molecules on commercial activated carbons and synthesized porous t-BN are systematically performed and compared regarding their adsorption performance. In addition, oxidation stability is tested, which is a critical parameter for activated carbons and can lead to adsorber fires, especially when ketones are adsorbed.

## 2 Methods and Materials

### 2.1 Material Characterization

The X-ray diffraction (XRD) analysis was carried out with a STOE STADIP device (STOE and Cie GmbH). A CuKα (40 kV, 40 mA) radiation and a Mythen 1K detector (DECTRIS) were used. The step width was set to 0.2°2θ s^–1^.

An Alpha II FT-IR spectrometer from Bruker, equipped with a platinum ATR module (diamond crystal) was used for ATR-IR measurements. Before the analysis approximately 5–10 mg of the powdered sample was put on the crystal. A resolution of 4 cm^–1^ and a range between 400 to 4000 cm^–1^ was chosen. Each scan was repeated 20 times.

All adsorbents were characterized by methods developed for activated carbons.^42^ An autosorb iQ3 (Quantachrome Instruments) was used to measure nitrogen sorption isotherms at 77 K. For the adsorption tests, 0.6 g of the activated carbons and 0.5 g of the boron nitrides were used. Prior to each measurement the samples were outgassed for 6 h under vacuum at 150 °C. Outgassing was considered sufficient once the pressure increase was less than 25 mTorr min^–1^. Pore size distributions were determined using QSDFT for the adsorption branch of nitrogen sorption at 77 K on carbon with slit and cylindrical pores. The total pore volume as well as the micropore volume for pores ≥2 nm were determined from the pore size distribution. DIN ISO 9277^43^ was used to determine the BET (Brunauer-Emmet-Teller) surface areas.^44^

SIT and PIO are calculated thermogravimetrically in accordance to Suzin et al.^41^ For the analysis, a Discovery TGA 550 (TA Instruments) was used. Prior to analysis, the samples were conditioned at 150 °C in nitrogen atmosphere (purity 4.5). At first, the samples were measured in nitrogen with a heating rate of 5 K min^–1^. After that, a second identical measurement in synthetic air (Linde Gas) was carried out. The thermogram was calculated by the difference between both measurements. From the thermogram, the SIT can be determined by applying a tangent to the inflection point. Its intersection with the baseline (100% weight) is the SIT. The PIO is defined as the first temperature at which the mass of the sample changes by at least 0.01% compared to the mean value of the previous five measuring points.

**Figure 1:**
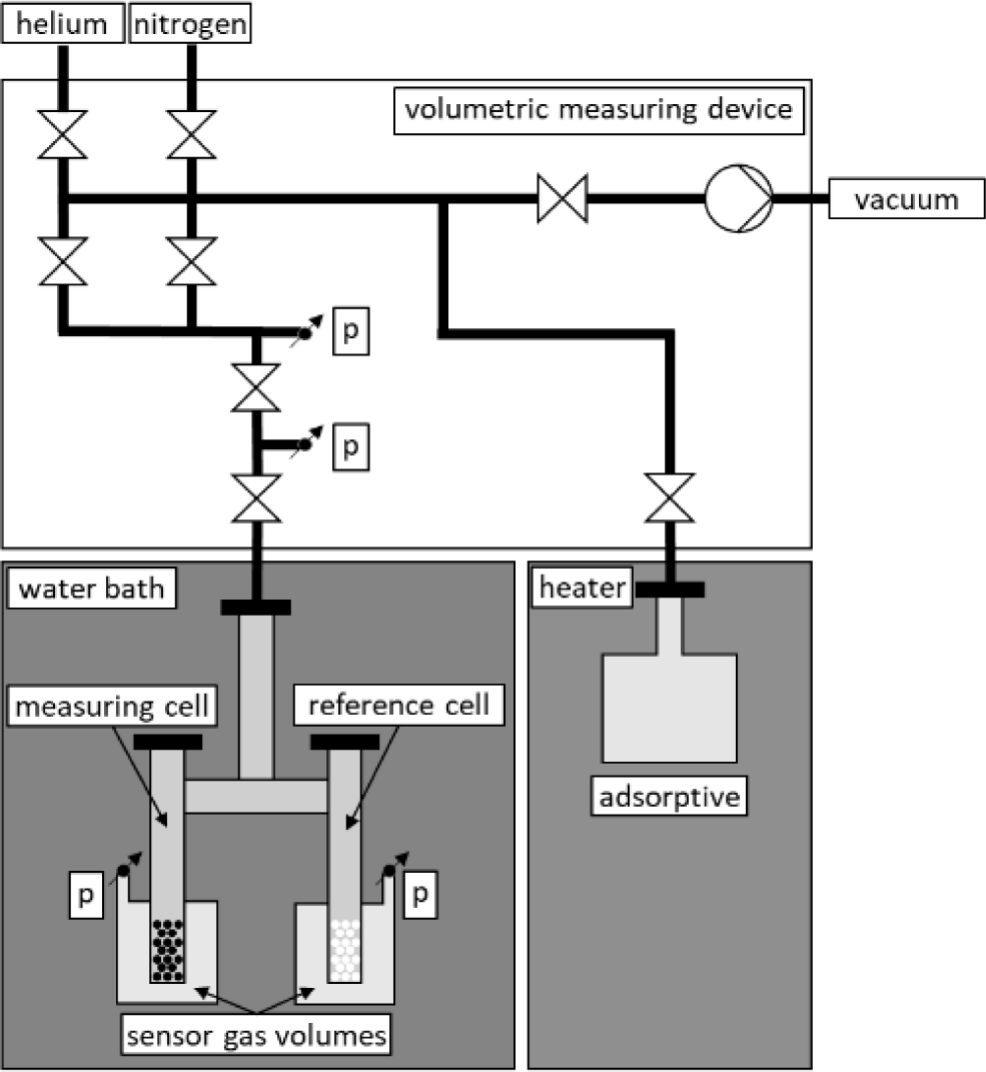
Schematic representation of the experimental setup reproduced from.^47^ Copyright 2022 American Chemical Society.

A sensor gas calorimeter is used with a commercially available volumetric adsorption instrument (BELSORP-max, BEL JAPAN, INC.) to determine the load-dependent heat of adsorption. The adsorption isotherms are measured cumulatively using the volumetric device between high vacuum (10^–3^ Pa) and ambient pressure (101.3 kPa). While the pressure of gaseous adsorbents is adjusted by a pressure reducer on the gas cylinder, liquid components are dosed by evaporation in the head of the BELSORP-max at 40 °C. The liquid adsorptive is dosed into the manifold of the measuring device by opening a valve, which releases the saturated gas phase into an evacuated dosing section. A defined quantity of the adsorptive can be dosed into the sample vessel using a further valve. If no further dosing is required, the valve is closed and no adsorptive escapes from the vessel. For the calorimetric measurement setup, two cells, one measurement and one reference cell, are constructed symmetrically and connected to each other. The inner volume of the measuring cell is filled with adsorbent and the reference cell with inert glass beads. Connected to pressure sensors, the outer volumes are filled with a sensor gas. During adsorption, heat is released in the measuring cell. The heat flow induced to the sensor gas volume leads to a temperature and thus to a pressure increase. In the reference cell no adsorption takes place resulting in a constant pressure of the sensor gas volume. The time-dependent pressure difference is used as a calorimetric signal to determine the heat of adsorption. The schematic setup is shown in Figure 1. A more detailed description of the experimental approach and the calculation, the reader is referred to Bläker et al.,^45^ Mauer et al.^46^ and Gehrke et al.^47^ For each measurement 0.6 g of the activated carbon D47 and 0.5 g of the BN-meso was used. The sample preparation is identical to that for the nitrogen sorption measurements at the autosorb iQ3.

Adsorption isotherms of various hydrocarbons were measured gravimetrically in a magnetic suspension balance (Rubotherm). While the concentrations and partial pressures of gaseous adsorptives and the nitrogen carrier gas flow can be adjusted via mass flow controllers (MFC), liquid components are dosed by evaporation in a temperature-controlled magnetic suspension balance and diffusion into a nitrogen carrier flow. The concentration and thus the partial pressure can be precisely determined from the time-dependent mass change of the balance. If no dosing is required, the valves upstream and downstream of the magnetic suspension balance are closed and the adsorptive cannot escape. The adsorption measurements were carried out after degassing 25 mg of the adsorbent in a vacuum at 150 °C for 3 h. After cooling down to a temperature of 25 °C, the adsorptive was added to the gas phase until the desired pressure was reached. Low pressures between 0.05 to 0.95 mbar were used in order to investigate interactions between the BN surface and the adsorptive in the monolayer adsorption region with minimal lateral interactions between adsorbed molecules. The isotherms were measured cumulatively with equilibrium points at partial pressures of 0.05, 0.15, 0.25, 0.35, 0.45, 0.55, 0.65, 0.75, 0.85, and 0.95. Adsorption equilibrium was assumed for each equilibrium step once the change in the measurement signal over 20 min was less than 0.1% by weight. To account for the change of gas phase density with varying pressures and compositions for each adsorptive a calibration curve was measured prior to the adsorption experiments and subtracted from the measured data. The experimental error of loading calculated using Gaussian error propagation is 6 to 10%.

Water-vapor adsorption isotherms were measured volumetrically with an Autosorb iQ3 (Quantachrome Instruments) at 25 °C. High-purity water with an electrical resistance of 18.2 MΩ cm was used. The measurement was applied at relative pressures of 0.0 < p p_0_^–1^ < 0.9. The sample preparation prior to the measurement was carried out as explained for nitrogen sorption. For each measurement 0.6 g of the activated carbon and 0.5 g of the BN-meso was used.

### 2.2 Adsorptives

As adsorptives, propane (99.5%, Air Liquide), propanal (>99%, Acros Organics), acetone (100%, VWR), *n*-hexane (99.3%, VWR), and toluene (100%, VWR) were used. Important properties of the adsorptives are shown in Table 1. Propane and *n*-hexane were chosen to represent nonpolar alkanes, where induction and dispersion interactions are expected. As polar adsorptives, propanal as an aldehyde and acetone as a ketone were picked due to their similar properties except the dipole moment. The higher electronegativity of the oxygen atom compared to the carbon atom of the carbonyl respectively aldehyde group shifts electron density to the oxygen atom, which leads to a negative partial charge on the oxygen atom and a positive partial charge on the neighboring carbon atom. Consequently, dipole–multipole interactions are possible. In addition, dispersion interactions can be formed with the CH_3_ respectively CH_2_ groups. As a result of sp^2^ hybridization, the aromatic toluene has a delocalized π-electron system with a negatively charged electron cloud above and below the positively charged hexagonal carbon ring, resulting in a quadrupole moment of (33.3 ± 2.1) 10^–40^ C m^2^.^48^ Hence, toluene can form primarily quadrupole–multipole and quadrupole-induced multipole interactions. With a delocalized π-electron system, π–π interactions are also possible.^49^

**Table 1:** Adsorptive Properties^48,50,51,52,53^

	Molar mass in mol g^–1^	Vapor pressure (25 °C)in mbar	Polarization volume in 10^–30^ m^3^	Dipole moment in D	Enthalpy of evaporation in kJ mol^–1^
propane	44.10	9776	6.29	0.08	16.3
propanal	58.08	423	6.50	2.72	29.7
acetone	58.08	308	6.33	3.44	31.3
*n*-hexane	86.18	202	11.90	0.08	31.0
toluene	92.14	38	12.30	0.38	37.3

### 2.3 Activated Carbons

**Figure 2:**
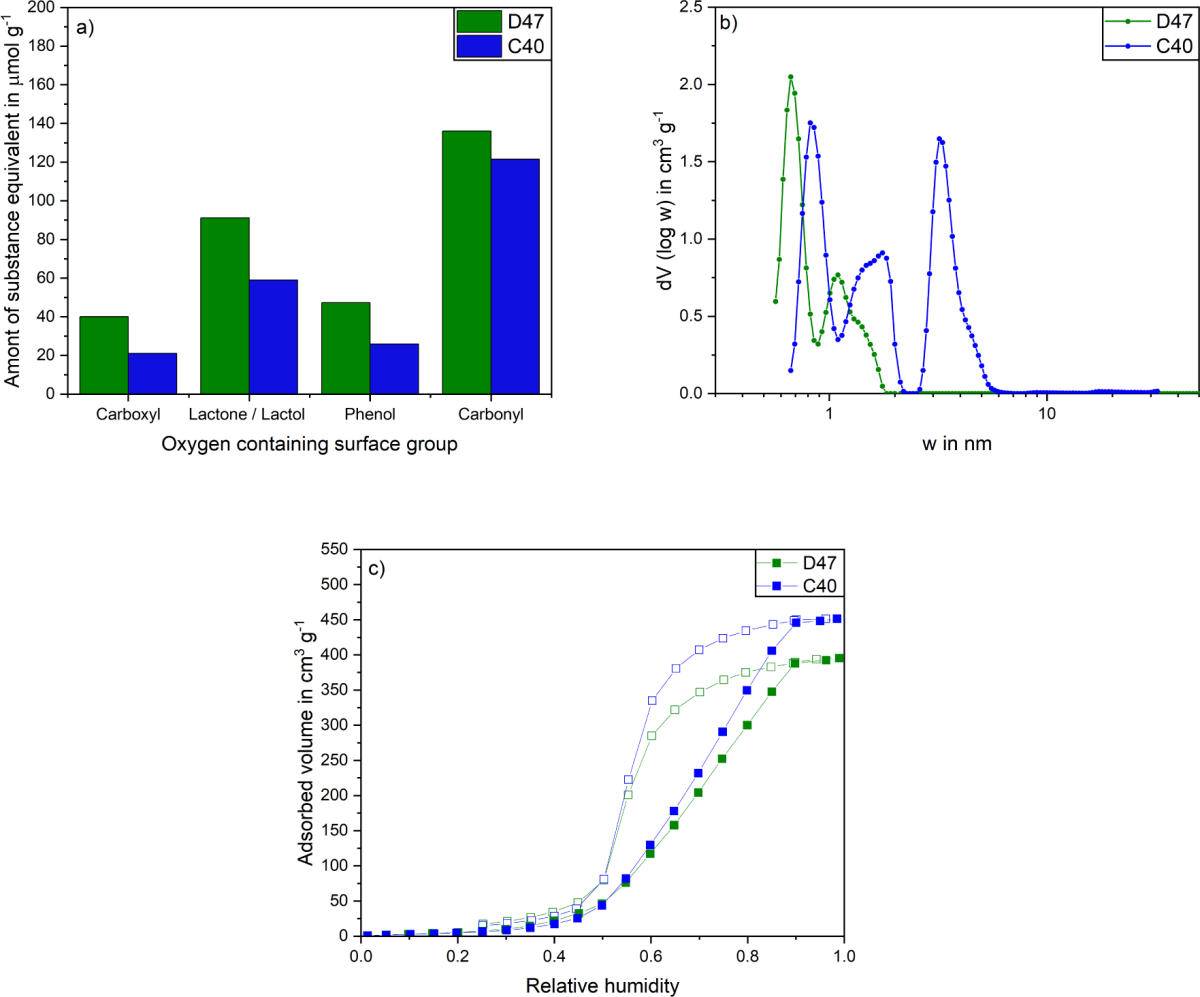
a) Acidic oxidic surface groups, b) pore size distributions, and c) water-vapor isotherms on activated carbons D47 (green) and C40 (blue). Pore size distribution of D47 reproduced from.^54^ Copyright 2022 American Chemical Society.

In this study, two hard coal-based activated carbons D47/3-Extra (CarboTech GmbH, Essen), referred to as D47, and C40/4-Extra (CarboTech GmbH, Essen), referred to as C40, were chosen. The two steam-activated samples were activated for different times, resulting in different porosities. The activated carbons have already been used in a previous study for adsorption of inhalation anesthetics in humid atmosphere and are therefore well-known.^54^ The composition of both activated carbons was determined by elemental analysis according to DIN EN ISO/IEC17025:2005 (EUROEA3000 Elemental Analyzer, EuroVector S.p.A.).^55^ D47 has a composition of 88.9% C, 0.6% H, 5.7% O and 4.6% ash and C40 a composition of 90.5% C, 0.6% H, 3.5% O and 4.6% ash. The lower oxygen content suggests that C40 is less polar. However, since the elemental analysis data cannot be used to determine whether the atoms are located within the activated carbon lattice or on the surface, Boehm titration^33^ measurements of D47 and C40 were carried out. Using Boehm titration the substance equivalents of the acidic oxidic surface groups carboxyl, lactone/lactol, phenol and carbonyl can be determined. The results of the Boehm titration are shown in Figure 2a. Even if the number of all acidic oxidic surface groups is slightly higher for the C40 than for the D47, the values for both activated carbons are in the lower range compared to conventional activated carbons.^33^ Therefore, a mainly nonpolar surface with a low number of active centers can be assumed for the C40 and D47.

The pore size distribution of both materials is shown in Figure 2b. D47 has a microporous pore size distribution with no mesopores. Due to a longer activation time, the C40 also has mesopores, resulting in a much lower proportion of micropores. The BET surface areas and pore volumes of both materials are shown in Table 2. C40 has a much higher BET surface area and pore volume than D47, due to the longer activation time. The micropore volume of both activated carbons is very similar.

**Table 2:** Specific Surface Area and Volumes of Both Activated Carbons^a^

	BET surface area in m^2^ g^–1^	Micropore surface area in m^2^ g^–1^	Total pore volume| in cm^3^ g^–1^	Micropore volume in cm^3^ g^–1^
D47	1000	914	0.38	0.38
C40	1200	750	0.54	0.41

a Surface areas and pore volumes of D47 reproduced from.^54^ Copyright 2022 American Chemical Society.

To investigate the influence of the structural properties and the surface chemistry on the adsorptive properties, water-vapor isotherms were measured on both activated carbons (see Figure 2c)). Both isotherms show a typical type V curve for nonpolar activated carbons. The small increase at low partial pressure is due to the low number of polar surface groups, so that predominantly weak induction interactions are formed with the nonpolar activated carbon surface. With increasing relative pressure, the water molecules form lateral interactions and water clusters are formed. From a partial pressure of around 0.5, pore condensation begins, which leads to a significant increase in loading. Due to the higher porosity, the C40 has a higher saturation load.

### 2.4 Porous Boron Nitrides

In the present work, a primary mesoporous t-BN, referred to as BN-meso is investigated. A detailed analysis of the materials properties and water stability is given in a previous work.^20^ BN-meso was synthesized from a precursor mixture of urea (Alfa Aesar, > 98%) and boric acid (VWR Chemicals, > 99%). Before heat treatment, both precursors were mechanically homogenized in a 3:1 molar ratio with a ball mill (PM 100, Retsch 450). After that, a heat treatment with two heating levels was applied in a tube furnace (ROC 50/610/14, Thermconcept) in nitrogen flow (80 L h^–1^, purity 5.0). The first temperature plateau was set at 200 °C for 2 h, and the second plateau at 1300 °C for 4 h. In general, a heating ramp of 5 K min^–1^ was chosen.

By using two temperature plateaus, one in the range of precursor decomposition, and one at high crystallization temperatures, we were able to synthesize a highly mesoporous BN with high crystallinity.^56^ Compared to other microporous BNs BN-meso is largely water-vapor stable and hence suited for applications in adsorption.^20^ However, due to the higher crystallinity, the material has a relatively low BET surface area of 214 m^2^ g^–1^ (Table 3). To further increase the BET surface area of BN-meso we used an acidic treatment to remove residual boron oxide and amorphous BN as described by Shankar et al.^21^ The acidic treatment was carried out for 7 days with 0.1 molar HCl (Roth, 37%) in a PP-beaker (0.5 g BN in 125 mL HCl). After that, the material was washed three times with 50 mL water and dried at 80 °C for 24 h. The acidic-leached sample is referred to as BN-meso-Leach in the following.

**Table 3:** BET Surface Areas and Pore Volumes of Both Boron Nitrides^a^

	BET surface area in m^2^ g^–1^	Micropore surface area in m^2^ g^–1^	Total pore volume in cm^3^ g^–1^	Micropore volume in cm^3^ g^–1^
BN-meso-Leach	366	106	0.57	0.06
BN-meso	214	55	0.51	0.03

a Surface areas and pore volumes of BN-meso reproduced from refs (20),^56^ Copyright 2022 Elsevier B.V.

**Figure 3:**
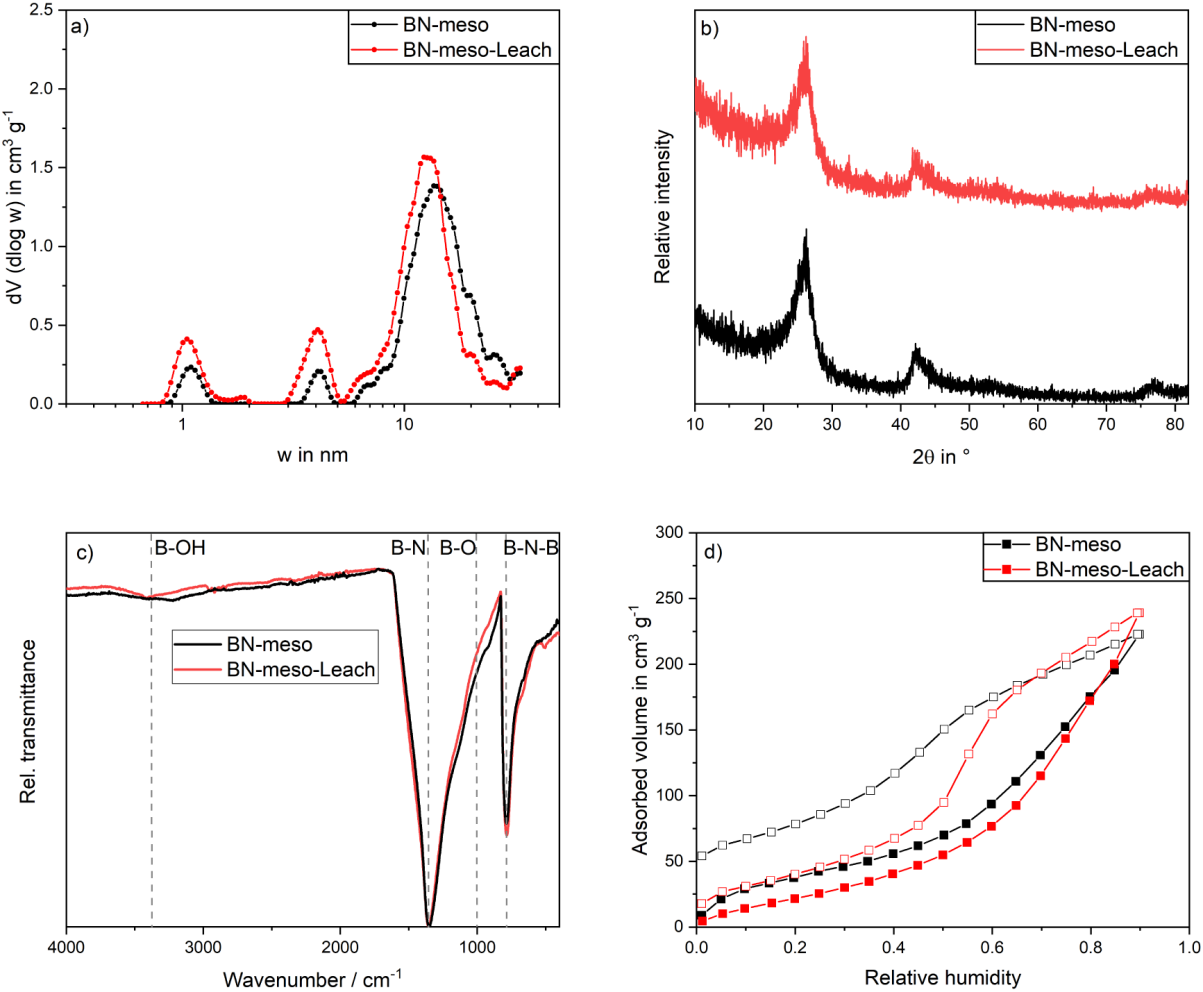
a) Pore size distributions, b) XRD patterns, c) ATR-IR measurement, and d) water-vapor isotherms of BN-meso (black) and BN-meso-Leach (red). Pore size distribution, XRD, ATR-IR spectrum, and water-vapor adsorption isotherm of BN-meso reproduced from.^20^ Copyright 2022 Elsevier B.V.

As shown in Table 3, the porosity of BN-meso was increased by leaching. The BET surface area increased by ∼71% and the pore volume by ∼12%. The micropore volume determined by the pore size distribution shows good agreement with the values determined according to DIN 66 135^57^ with the t-method by Lippens and de Boer.^47^ The increase in porosity by leaching can be attributed either to the destruction of the boron nitride framework and the associated generation of defects or to the leaching of unreacted educts, like boron oxide. To observe, if the acidic leaching led to changes in the morphology, scanning electron microscopy (SEM) analysis was performed. The results are given in Figure S1. As depicted in the SEM images both materials show similar flake-like structures without significant differences after the treatment. Hence, no ongoing decomposition of the material is indicated. To observe changes in the micro- and mesopore structure nitrogen sorption was used. Figure 3a shows the pore size distributions of the two boron nitrides. It can be seen that BN-meso-Leach has a higher differential volume, although there is no shift in the pore size. The similar pore size distribution indicates that no new pores were formed. Instead, the increase in pore volume at similar pore sizes indicates to the opening of previously blocked pores. This observation is in alignment with previous studies, where an initial increase in pore volume and specific surface area after water exposure was attributed to the removal of unreacted boron oxide.^20,21^

To further identify if the acidic treatment led to any changes, XRD and ATR-IR analysis was performed. In the XRD analysis (Figure 3b) three distinct reflexes at 2θ = 26°, 2θ = 43° and 2θ = 76° are observed for both materials. The reflexes can be assigned to h-BN. Due to the broad reflex widths, a turbostratic structure is implied. By comparing the XRD patterns before and after the acidic treatment, no changes can be observed. Therefore, no reaction of amorphous or turbostratic BN with water is assumed as in this case a decrease in reflex width or the formation of new boron oxide reflexes after the treatment would be expected.^21^ This conclusion is further reinforced by ATR-IR analysis. As shown in Figure 3c both materials have distinct bands at ∼1360 cm^–1^ and ∼800 cm^–1^ that can be attributed to B–N stretching and B–N–B bending vibrations.^58^ After acid treatment, no new bands are formed. On the contrary, a slight decrease in the B–O shoulder at ∼1000 cm^–1^ can be observed, indicating the removal of boron oxide or a reduction in B–O defect sites.^59^

Based on these findings, it is assumed that the acidic treatment does not lead to a strong reaction of boron nitride with water, but instead to the removal of amorphous boron oxide residues which results in a pore opening.

In Figure 3d, the water-vapor adsorption of BN-meso and BN-meso-Leach is shown. Both isotherms can roughly be classified to be Type IV(a) with an H5 hysteresis loop. While BN-meso shows a steep uptake at low partial pressures, indicating a hydrophilic surface or chemical surface reactions with water,^20^ the less pronounced slope of the BN-meso-Leach indicates either a more hydrophobic surface or less chemical surface reactions. In addition, it is evident that BN-meso-Leach shows a much smaller opening of the hysteresis at low relative pressures of p p_0_^–1^ ≈ 0. In general, the size of the opening is an indication of chemisorptive, nonreversible adsorption processes. While the large hysteresis opening of BN-meso indicates a pronounced chemical reaction of the adsorbed water, the smaller hysteresis opening of BN-meso-Leach implies a higher water-vapor stability after the acidic treatment. It can also be observed that the adsorption capacity on both boron nitrides is comparable over the entire relative pressure range up to p p_0_^–1^ ≈ 0.9. Thus, a higher number of preferred adsorption sites for water and a more hydrophobic surface by leaching is not detectable. This assumption is reinforced by the ATR-IR measurements, where no significant changes in the B–OH and B–O bands were depicted. After water-vapor adsorption, nitrogen sorption was measured on the BN-meso-Leach. The nitrogen sorption isotherms before and after water-vapor adsorption are depicted in Figure S2. The two measurements show similar curves with only slightly different nitrogen capacities and thus confirm the presumed higher resistance of the BN-meso-Leach toward water.

## 3 Results and Discussion

### 3.1 Adsorption Isotherms of Hydrocarbons

**Figure 4:**
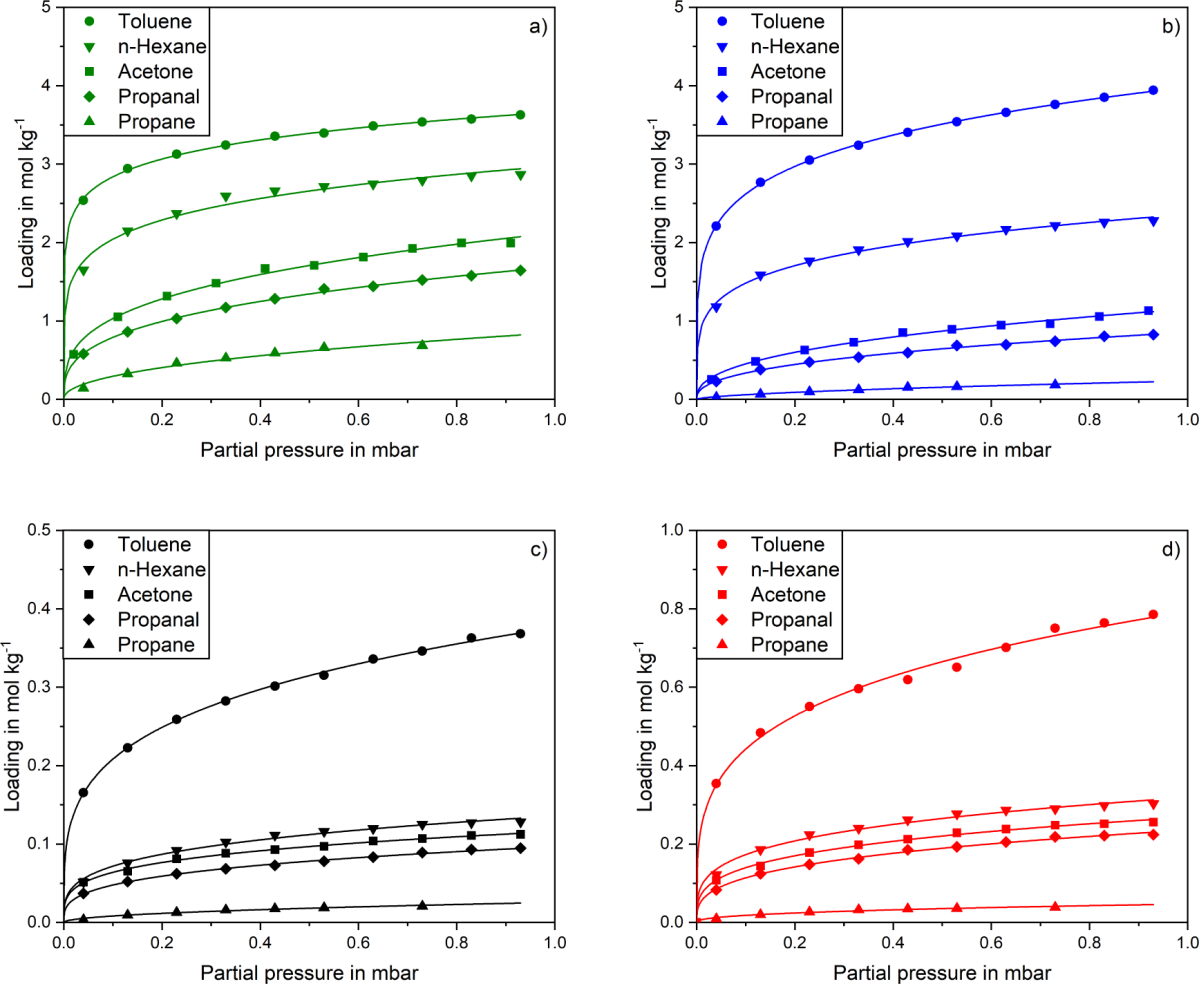
Adsorption isotherms of toluene, *n*-hexane, acetone, propanal, and propane on a) activated carbon D47, b) activated carbon C40, c) BN-meso and d) BN-meso-Leach at 25 °C.

Figure 4 shows adsorption isotherms of various hydrocarbons on activated carbons D47 and C40 as well as boron nitrides BN-meso and BN-meso-Leach. Based on the discussion of the adsorption properties on well-known activated carbons, the adsorption properties of the synthesized t-BNs are classified and evaluated with regard to their use in gas-phase applications. In the following diagrams, Freundlich isotherms are fitted to the measured data. The fitted Freundlich parameters of the isotherms are given in Table S1.

For the activated carbon D47 (Figure 4a), all isotherms arrange according to the saturation vapor pressure and molar mass of the adsorptives. Propane, with the highest saturation vapor pressure and lowest molar mass has the lowest loading, and toluene, with the lowest saturation vapor pressure and highest molar mass shows the highest loading. Despite similar polarization volumes of 6.29 to 6.50 × 10^–30^ m^3^, the three C_3_-hydrocarbons propane, propanal, and acetone have different loadings. This variation is caused by their different polarities. Propane is nonpolar and has no significant dipole moment of 0.08 D. In comparison, propanal and acetone are polar with dipole moments of 2.72 and 3.44 D. Similar to nonpolar propane, the polar molecules can form dispersion interactions of the CH_3_ respectively CH_2_ groups with the activated carbon. The improved adsorption of the polar molecules results from the additional dipole–dipole interactions between the partially negatively charged oxygen atom of the molecules with the positively charged hydrogen atoms of the functional surface groups (carboxyl, lactone, lactol, phenol, or carbonyl groups) and the partially positively charged carbon atom with the negative charge of the delocalized π-electron system as well as the negatively charged oxygen atoms of the functional surface groups. *n*-hexane has higher loadings than the C_3_-hydrocarbons which can be attributed to the doubled amount of interaction sites and the much higher polarization volume. Hence, stronger induction and dispersion interactions occur. While the alkanes probably adsorb parallel to the surface to maximize the contact area, an angled arrangement to the functional surface groups or a planar arrangement to the graphite surface is possible for the polar adsorptives.

During adsorption of toluene various overlapping interactions are possible. The quadrupole of the toluene can induce a multipole in the graphitic areas, resulting in a quadrupole-induced multipole interaction. As a result of the high polarization volume, a temporary multipole can also be induced in the toluene and dispersion interactions can be formed with the activated carbon. In addition, multipole–multipole interactions can be formed with the oxygen-containing polar surface groups. In this case, the negatively charged electron cloud of toluene can interact with the positively charged hydrogen atoms of the functional surface groups and the positively charged hexagonal ring of toluene can interact with the negatively charged oxygen atoms of the functional surface groups. Another interaction mechanism is the π–π interaction between the delocalized π-electron system of toluene and the graphitic regions of the activated carbons. However, the mechanism is not yet fully understood.^49^ While off-center parallel stacking or edge-to-face interaction is preferred due to the π–π interactions,^49^ an angled arrangement to the functional surface groups is likely. However, due to the superposition of the various interactions, it is not possible to make a clear statement about the spatial arrangement of toluene molecules on an activated carbon surface.

The activated carbon C40 in Figure 4b shows a similar adsorption behavior as D47, with the same arrangement of the isotherms. By comparing the results to those of D47, it can be observed that the loadings for the C_3_-hydrocarbons are lower for C40, but the loadings of *n*-hexane and toluene are slightly higher. Since both activated carbons have similar densities and surface groups (as shown in Figure 2, the different adsorption capacities can be related to their different pore size distributions. For adsorption of smaller C_3_-hydrocarbons smaller micropores, like for D47, are more preferred due to stronger induction and dispersion interactions. However, for larger molecules like *n*-hexane and toluene, larger pores are more beneficial due to sterical hindrance in very small pores.

The adsorption of hydrocarbons on BN-meso is shown in Figure 4c. All samples align in the same order as for activated carbons. However, the distances between the isotherms are different, which can be attributed to a different type or strength of the adsorption mechanisms. Adsorption of the nonpolar alkanes is largely determined by the induction of CH_3_ and CH_2_ groups by the boron and nitrogen atoms. The alkanes will probably adsorb parallel to the surface. The larger distance of the isotherms of the polar adsorptives to propane and similar loadings as *n*-hexane indicate strong dipole–dipole interactions with polar h-BN layers. We assume, that those are further enhanced by defects within the layers, where reactive centers for polar molecules are formed.^60,61^ The best spatial arrangement is achieved when the distance between the carbonyl group of the acetone respectively the aldehyde group of the propanal to the boron nitride surface is minimized. Consequently, the molecules are probably arranged at an angle or perpendicularly.

During adsorption of toluene, quadrupole–dipole interactions can form between the negatively charged electron cloud of the toluene and the positively charged boron atoms as well as between the positively charged hexagonal ring and the negatively charged nitrogen atoms. The high loading of toluene compared to *n*-hexane can be attributed to the high polarity of boron nitride. The energetically best arrangement of the toluene on the boron nitride surface will probably be either a parallel or a perpendicular arrangement.

For BN-meso-Leach no changes in the arrangement of the isotherms can be observed compared to the previous findings (Figure 4d). The main difference between the adsorption of hydrocarbons on BN-meso and BN-meso-Leach is the higher loading of the hydrocarbons on the leached sample. We assume that BN-meso-Leach offers more reactive sites due to its higher porosity after the acidic treatment.

If the loadings of all adsorbents are compared, it becomes evident that the synthesized porous BNs show much lower loadings than the activated carbons. However, since those findings are related to the adsorbent mass this effect can be attributed to the much higher porosity of the activated carbons. As the adsorption measurements were measured for trace level concentrations of up to 1 mbar, micropores were almost exclusively filled. Due to the significant differences in pore size distribution, all loadings were normalized to the respective micropore surface area. In this way, the influence of pore size distribution can be largely eliminated and the influence of surface chemistry on adsorption can be discussed in detail.

**Figure 5:**
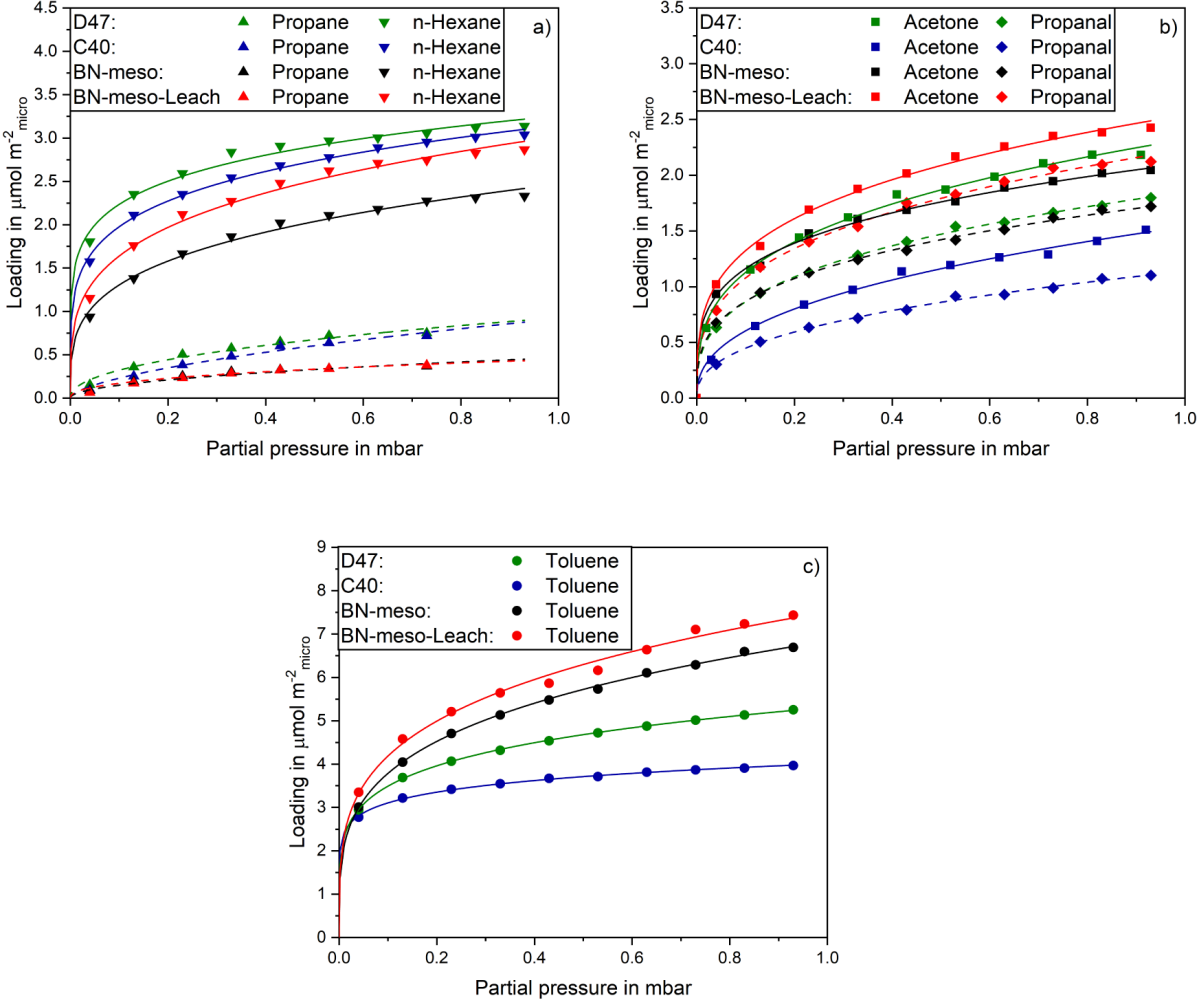
Loadings normalized to micro pore area of a) nonpolar propane and *n*-hexane, b) polar acetone and propanal and c) aromatic toluene on activated carbons and boron nitrides at 25 °C.

Figure 5 displays the normalized loadings, expressed in μmol m^2^_micro_, of the nonpolar alkanes propane and *n*-hexane, the polar acetone and propanal as well as the aromatic toluene on all samples. The fitted Freundlich parameters of the normalized isotherms are listed in Table S2. The normalized loadings of the nonpolar adsorptives (Figure 5a) on the activated carbons are higher than on the BNs. This underlines the assumption that the adsorption of alkanes is mainly due to dispersion interactions and that there is a more favorable arrangement in the micropores due to the smaller pore widths. As D47 has smaller micropores than C40, stronger dispersion interactions occur which leads to higher adsorptive loadings. The differences in loading of *n*-hexane for BN-meso and BN-meso-Leach cannot be explained by their porosity, as both samples have identical pore widths (see Figure 2) and adsorption occurs in similar pore geometries. The more favorable adsorption on BN-meso-Leach must therefore be due to a higher polarity, which leads to stronger induction interactions with the nonpolar alkanes and higher loadings.

The normalized loadings of the polar adsorptives acetone and propanal are shown in Figure 5b. Here, the activated carbon C40 has the lowest loading. D47 shows similar adsorptive loadings to BN-meso. The highest loadings are depicted by BN-meso-Leach. Since the normalization to the micropore surface has reversed the order of the loadings and the boron nitrides show higher loadings than the activated carbons, the surface chemistry of the boron nitrides is more favorable for adsorption of polar adsorptives. The more polar surface of the boron nitrides leads to stronger dipole–dipole interactions with the boron and nitrogen atoms compared to the multipole–multipole interactions with the hydrogen and oxygen atoms of the functional surface groups of the activated carbons. The comparable loadings of D47 and the BN-meso, despite their different surface chemistry, can be attributed to the different pore size distributions. As mentioned previously D47 has small micropores, resulting in a large number of attractive adsorption sites which compensate for the weaker dipole–dipole interactions with the adsorptives. The pore size distribution of the C40 is shifted toward larger pore widths compared to D47, so that compensation of the stronger dipole interactions is not possible, resulting in lower loadings result.

The normalized adsorption isotherms of toluene are shown in Figure 5c. BN-meso-Leach has slightly higher loadings than BN-meso which can be attributed to its higher polarity. For the activated carbons the different pore geometry of D47 compared to C40 leads again to slightly higher loadings for D47. In comparison, the BN materials depict higher adsorptive loadings than the activated carbons. In analogy to the polar adsorptives, this can be attributed to the higher polarity of the boron nitrides, resulting in stronger quadrupole–multipole interactions. The low normalized loadings on the activated carbons also lead to the conclusion that the strength of the π–π interactions plays a subordinate role.

In summary, if the loading is related to adsorbent mass the synthesized water-vapor stable porous BNs are not competitive with conventional activated carbons in volatile organic compounds (VOC) adsorption. Here, a low BET surface area and a low percentage of micropores are key factors. However, when the loading is normalized to the micropore surface area, characteristic properties of BN surfaces can be identified. After normalization, the BNs show much higher adsorptive loadings in the adsorption of polar and aromatic adsorbents than the activated carbons due to stronger adsorbent-adsorptive interactions.

### 3.2 Heats of Adsorption of Hydrocarbons

To quantitatively characterize the energetic surface properties of BN, volumetric-calorimetric adsorption measurements were carried out on BN-meso and systematically compared to those of activated carbon D47. While adsorption isotherms allow qualitative conclusions about the energetic value of adsorption sites, the heat of adsorption, which is a measure of the strength of the interactions, provides a quantitative analysis of the energetic value of adsorption sites. A higher heat of adsorption means stronger interactions. At low loadings, the adsorbent molecules occupy the energetically most valuable adsorption sites, where the interactions between the molecule and the adsorbent surface dominate. The more energetically heterogeneous the adsorbent is, the more the heat of adsorption decreases with increasing loading. In addition, interactions between the adsorbent molecules increase with increasing loading, resulting in a superposition of different adsorption mechanisms. Consequently, the initial heat of adsorption in the region of low loading is of particular interest, as this is where the energetically most valuable adsorption sites on the surface can be analyzed in detail.

**Figure 6:**
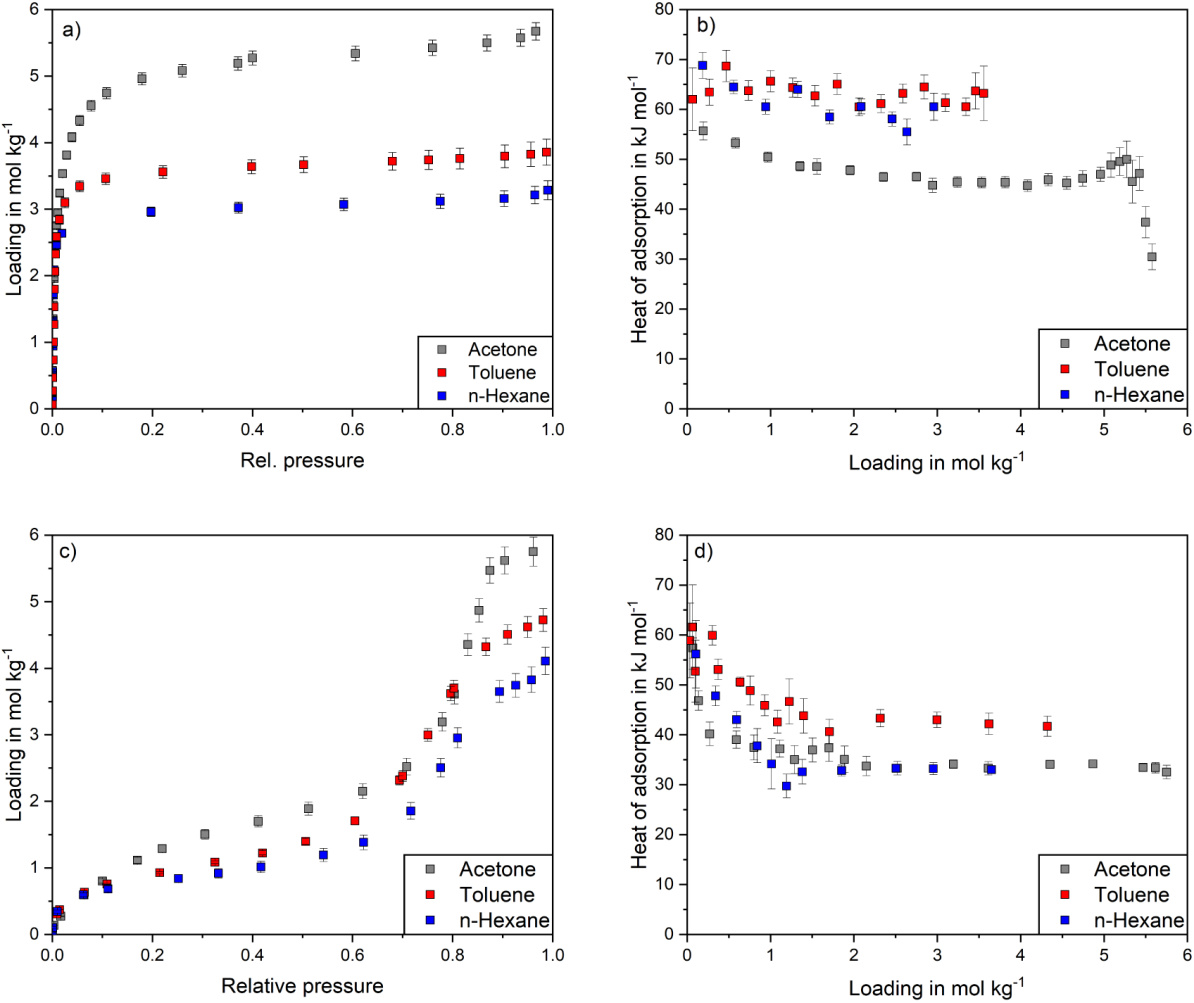
a) Adsorption isotherms and b) corresponding load-dependent heats of adsorption of acetone, toluene, and *n*-hexane on D47 as well as c) adsorption isotherms and d) load-dependent heats of adsorption on BN-meso.

Figure 6 depicts the a) adsorption isotherms and b) corresponding heats of adsorption of toluene, acetone and *n*-hexane on activated carbon D47 as well as c) adsorption isotherms and d) heats of adsorption on BN-meso. At low relative pressures all isotherms on D47 show a steep increase that changes with increasing pressure to a plateau-like progression. This type I isotherm is characteristic for microporous materials.^62^ In Figure 6b the load-dependent heats of adsorption are shown. Acetone has an initial heat of adsorption of 50 kJ mol^–1^. With increasing loading, the heat of adsorption decreases to 40 kJ mol^–1^ at 3 mmol g^–1^ until a plateau-like progression follows. Hence, for acetone, an energetically heterogeneous surface is observed. The high initial heat of adsorption can be explained by strong dipole–dipole interactions of acetone with polar functional surface groups (carboxyl-, lactone-, lactol-, phenol-, and carbonyl-groups) of D47394 Col:310. Since these surface groups have different polarities and accessibilities, interactions of different strengths are formed, which explain the decreasing heat of adsorption with increasing loading. Once all adsorption sites on the polar functional surface groups are occupied, dispersion and induction interactions with the graphite-like surface are predominantly formed.

Compared to acetone the heats of adsorption for toluene and *n*-hexane show higher values in the range of 50 to 60 kJ mol^–1^ throughout the entire measurement range. As the load-dependent decrease in heat of adsorption is negligible for those adsorptives the pore size distribution and the polar functional surface groups are expected to play only a minor role. It can be assumed that D47 has an energetically homogeneous surface for the adsorption of *n*-hexane and toluene. As already mentioned in Section 3.1, toluene can form both quadrupole–dipole and quadrupole-induced dipole interactions. But since the load-dependent heat of adsorption of toluene is nearly constant with increasing loading, it seems reasonable that rather quadrupole-induced dipole interactions are possible. Additional quadrupole–dipole interactions of toluene with the functional surface groups seem to have no significant influence on the adsorption. Both adsorptives have similar heats of adsorption and polarizabilities of 11.9 × 10^–30^ m^3^ (toluene) and 12.3 × 10^–30^ m^3^ (*n*-hexane), as well as weak dipole moments (Table 1). Therefore, similar induction and dispersion interactions with the activated carbon are expected. Induction and dispersion interactions are stronger for toluene and *n*-hexane than for acetone, due to the lower polarization volume of acetone with 6.33 × 10^–30^ m^3^. Therefore, the heat of adsorption of acetone is about 10 to 15 kJ mol^–1^ lower than for the other hydrocarbons.

On BN-meso, all adsorption isotherms show type IV shapes, that are characteristic for mesoporous adsorbents (see Figure 6c). Since BN-meso has a higher proportion of mesopores than D47, after monolayer adsorption and micropore filling, multilayer adsorption and capillary condensation occurs in the mesopores resulting in a second sharp increase in loading at relative pressures above 0.6. As shown in Figure 6d BN-meso exhibits similar initial heats of adsorption of 50 to 55 kJ mol^–1^ in case of all three hydrocarbons. The heats of adsorption decrease almost linearly up to a loading of 1.5 mmol g^–1^, after which constant values are achieved. Due to capillary condensation in the mesopores, lateral interactions are predominantly formed and the heat of adsorption corresponds to the enthalpy of vaporization. The decrease in the heat of adsorption of toluene is less pronounced compared to *n*-hexane and acetone, resulting in toluene having higher values over the entire loading range. This indicates a larger number of energetically favored adsorption sites with which quadrupole-dipole interactions are formed. The suspected higher polarity of BN is further underlined by the similar heats of adsorption of acetone and *n*-hexane, in contrast to adsorption on activated carbon. Due to the higher polarity of acetone, additional dipole–dipole interactions might compensate for the stronger induction and dispersion interactions formed with *n*-hexane which has a higher polarizability than acetone.

Compared to activated carbon D47, the initial heat of adsorption of acetone on BN-meso is higher, while toluene shows similar and *n*-hexane lower values. The higher initial heat of adsorption of acetone on BN-meso can be explained by stronger dipole–dipole interactions and thus confirms the previously assumed higher surface polarity of boron nitrides. However, due to the lower porosity of boron nitride compared to activated carbon, the heat of adsorption decreases for all adsorptives even at lower loadings.

### 3.3 Flammability and Oxidation Stability

**Figure 7:**
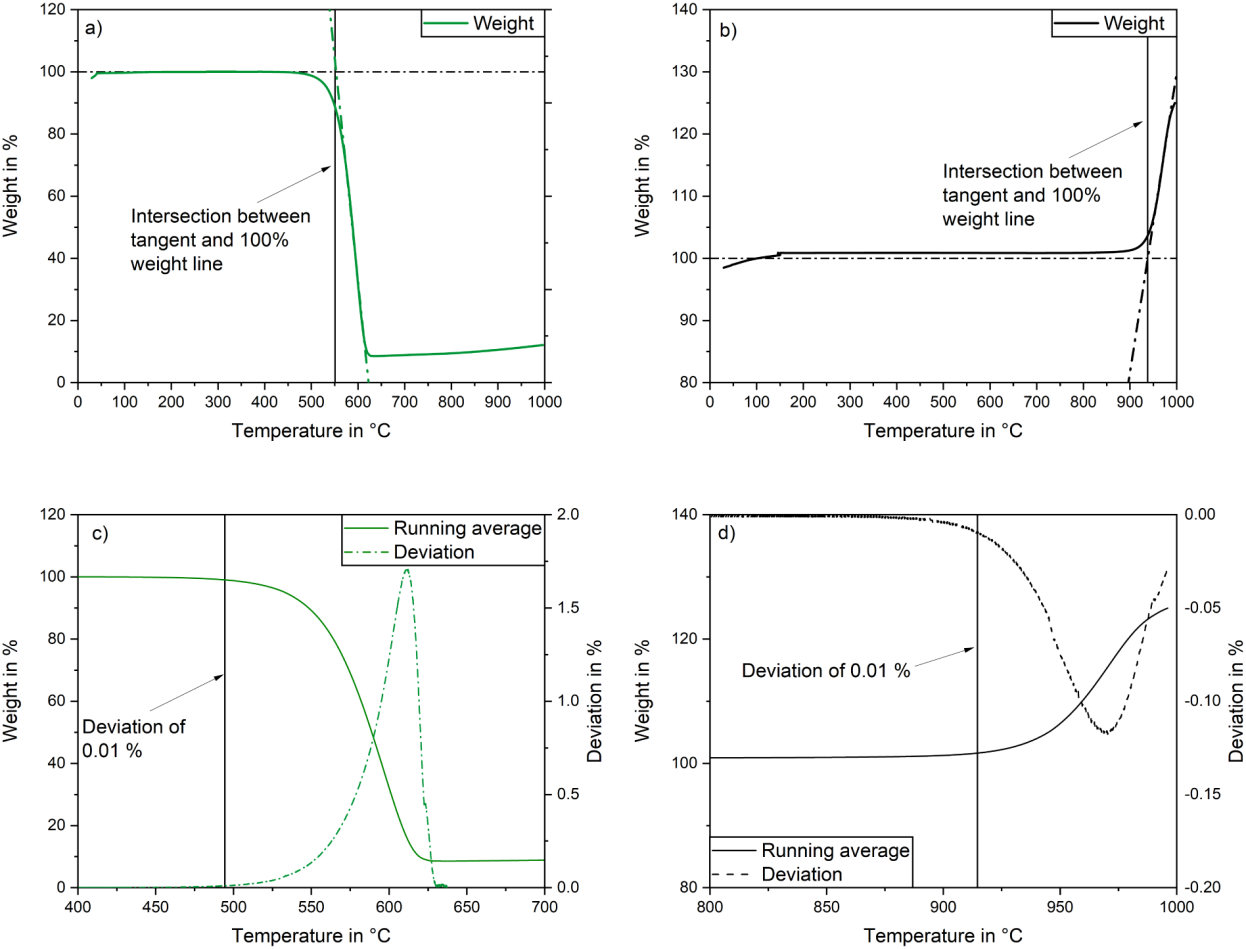
Thermograms of a) activated carbon D47 and b) boron nitride BN-meso with graphical determination of SIT as well as derivations of the thermograms of c) D47 and d) BN-meso with graphical determination of PIO.

Flammability and oxidation stability of adsorbents are important parameters for assessing whether these materials are suitable for technical processes. Especially activated carbons are subject to adsorber fire which must be considered in the design of adsorption processes and apparatuses. For the activated carbon D47 and the boron nitride BN-meso, the graphical determination of the SIT and the PIO are shown in Figure 7. The SIT and PIO plots for the activated carbon C40 and the boron nitride BN-meso-Leach are given in Figure S3 in the Supporting Information. To determine the PIO, a mass deviation of 0.01% compared to the mean value of the previous five measuring points was selected. This procedure is shown graphically by the running average and its deviation.

Table 4 gives an overview of the SIT and PIO of the four adsorbents. For activated carbons, the PIO is around 500 °C. At slightly higher temperatures of ∼550 °C combustion of the materials, indicated by a mass loss, starts. Thereby, the sample D47 which showed the best adsorption results has the lowest oxidation stability. For the BNs the PIO and SIT are much higher with temperatures above 900 °C. In contrast to activated carbons, during oxidation of BN, no mass loss occurs, because an oxidation reaction of BN and oxygen to nonvolatile and slightly heavier boron oxide takes place. Interestingly, the thermal stability of BN-meso-Leach is slightly higher than for BN-meso. This could be due to the removal of amorphous residuals during the acidic treatment.

**Table 4:** Spontaneous Ignition Temperature (SIT) and Point of Initial Oxidation (PIO) of the Adsorbents

	SIT in °C	PIO in °C
D47	553	494
C40	574	515
BN-meso	939	916
BN-meso-Leach	958	935

## 4 Conclusions

In the present work, the adsorption performance and the oxidation stability of two synthesized, water-vapor stable boron nitrides BN-meso and BN-meso-Leach were compared to two commercially available activated carbons D47 and C40. All adsorbents were extensively characterized. While the micropore volume of the activated carbons is almost ten times larger than that of that of the boron nitrides, the boron nitrides have slightly larger total pore volumes. Using elemental analysis and Boehm titration, it was shown that the activated carbons have a largely nonpolar surface with only a small number of acidic oxidic surface groups. Boron nitrides are assumed to have a higher polarity due to the electronegativity difference between the boron and nitrogen atoms. In order to analyze the boron nitrides in more detail and evaluate their suitability for use in technical adsorption processes, adsorption isotherms of the five hydrocarbons propane, propanal, acetone, *n*-hexane and toluene were measured in trace level and compared with measurements on the activated carbons. On all adsorbents, the isotherms align with increasing loading from propane to propanal, acetone, *n*-hexane and toluene. However, the activated carbons have up to ten times higher loadings for all adsorptives compared to the boron nitrides. This is mainly due to the higher microporosity, as the measurements were carried out in the trace range, in which adsorption mainly takes place in the micropores. By normalizing the adsorptive loadings to the micropore surface, it was shown that the loadings of the polar and aromatic adsorptives are higher on the polar BN materials, while the nonpolar adsorptives still show higher loadings on the activated carbons. By normalizing the adsorptive loadings on the micropore surface, the order of the isotherms of the polar and aromatic adsorptives has reversed, so that BN materials show about 50% higher loadings than the activated carbons. The nonpolar adsorbents, on the other hand, still show higher loadings on the activated carbons. The better suitability for the adsorption of polar and aromatic compounds confirms the presumed higher polarity of the BN materials, resulting in stronger dipole–dipole interactions with the boron and nitrogen atoms of the BN materials compared to the multipole–multipole interactions with the hydrogen and oxygen atoms of the functional surface groups of the activated carbons.

By applying coupled volumetric and calorimetric experiments on BN-meso and D47, a correlation between the loading and the pore volume was shown. BN-meso showed a higher initial heat of adsorption for acetone due to stronger dipole–dipole interactions of the aldehyde with the polar BN surface. While the initial heat of adsorption on both adsorbents was comparable for toluene, the activated carbon D47 featured higher heats of adsorption for *n*-hexane due to the higher microporosity and hence stronger induction and dispersion interactions with the pore walls. With increasing loading, D47 showed the highest heats of adsorption for all hydrocarbons, despite the differences in surface chemistry. Thus, we concluded that at low loadings, especially with polar and aromatic adsorptives, the surface chemistry of the adsorbents is decisive for adsorption, whereas with increasing loadings this influence decreases and the pore size distribution becomes the dominant influencing factor.

At last, the oxidation stabilities of the BNs were compared to those of the activated carbons. Thereby, a much higher PIO and SIT were observed for BN with oxidation temperatures above 900 °C.

Summarizing, it can be noted that at standard conditions the synthesized water-vapor stable BNs are no alternative to conventional activated carbons. However, due to the extremely high oxidation stability of the BN it could be used in high-temperature applications for example as catalyst supports. In the field of gas phase adsorption, mesoporous BN could also be used to separate polar and nonpolar adsorptives. However, in order to make porous BN accessible for industrial adsorption applications, a method must be found to increase the microporosity of the material without impairing its chemical and thermal stability. Since BN has advantageous surface properties, especially for the adsorption of polar and aromatic molecules, future work has to focus on optimizing porous BN for the adsorption of these adsorptives. The goal is to achieve loadings comparable to commercial activated carbons while maintaining the higher chemical and thermal stability.

## Data Availability

The data underlying this study are available in the published article and its online Supporting Information.

## Supporting Information Available

SEM images; Nitrogen adsorption and desorption isotherms (77 K) before and after water-adsorption desorption; SIT and PIO for C40 and BN-meso-Leach; Freundlich isotherm parameters and regression coefficients; acidic oxidic surface groups of D47 and C40; Pore size distribution data of D47, C40, BN-meso and BN-meso-Leach; water-vapor isotherm data of D47, C40, BN-meso and BN-meso-Leach; gravimetric adsorption isotherm data of toluene, *n*-hexane, acetone, propanal and propane on D47, C40, BN-meso and BN-meso-Leach; and volumetric adsorption isotherm as well as heat of adsorption data of acetone, toluene and *n*-hexane on D47 and BN meso (PDF)
XRD and IR data on BN-meso and BN-meso-Leach as well as thermo gravimetric data of D47, C40, BN-meso and BN-meso-Leach (XLSX)
