# Supplementary material for: Adsorbent Properties of Porous Boron Nitride and Activated Carbon: A Comparative Study
Source: ACS Omega. 2024 Oct 11;9(42):42721–33. doi: 10.1021/acsomega.4c02625 (PMC11500363; doi:10.1021/acsomega.4c02625)
Supplement: Supplementary file 1 — ao4c02625_si_001.pdf [file ao4c02625_si_001.pdf]

ACS Omega

Supporting Information

Adsorbent properties of porous boron nitride and activated carbon - a comparative study

Christian Bläker,[1,*] Tim Jähnichen,[2] Jan Hojak,[1] Laura Gehrke,[1] Christoph Pasel,[1] Thomas Paschke,[3] Frieder Dreisbach,[3] Dirk Enke,[2] and Dieter Bathen[1]

[1] Chair of Thermal Process Engineering, University of Duisburg-Essen, 47057 Duisburg, Germany
[2] Institute of Chemical Technology, Leipzig University, 04103 Leipzig, Germany
[3] TA Instruments - Waters GmbH, 32609 Hüllhorst, Germany

* Correspondence should be addressed to Christian Bläker: christian.blaeker@uni-due.de

The morphology was characterized by SEM. A Nova NanoLab200 (FEI Company) equipped with an Everhart–Thornley detector was used. A voltage of 15 kV and a measuring distance of ~6 mm was set. Before measurement, the samples were sputtered with gold.

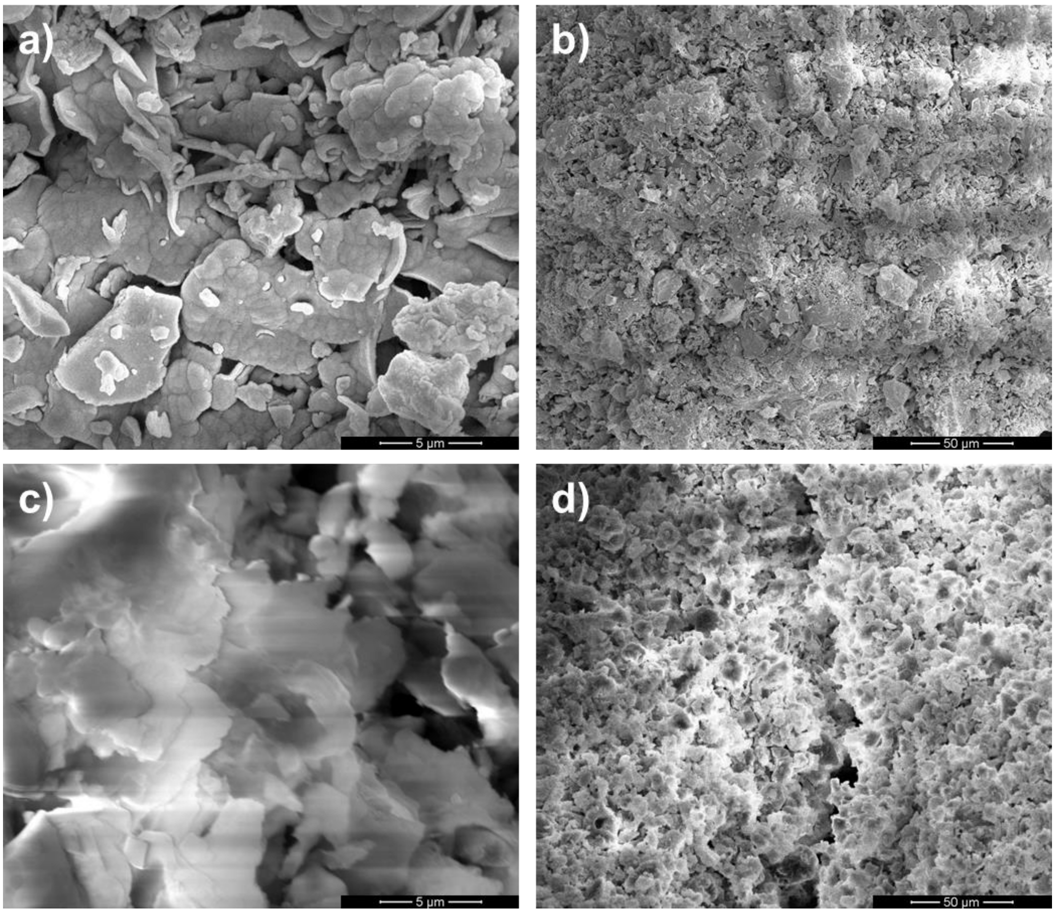

Figure S1: SEM images of a), b) BN-meso (ground) and c), d) BN-meso-Leach at different magnifications. Scale bars are a), c) 5 µm and b), d) 50 µm.

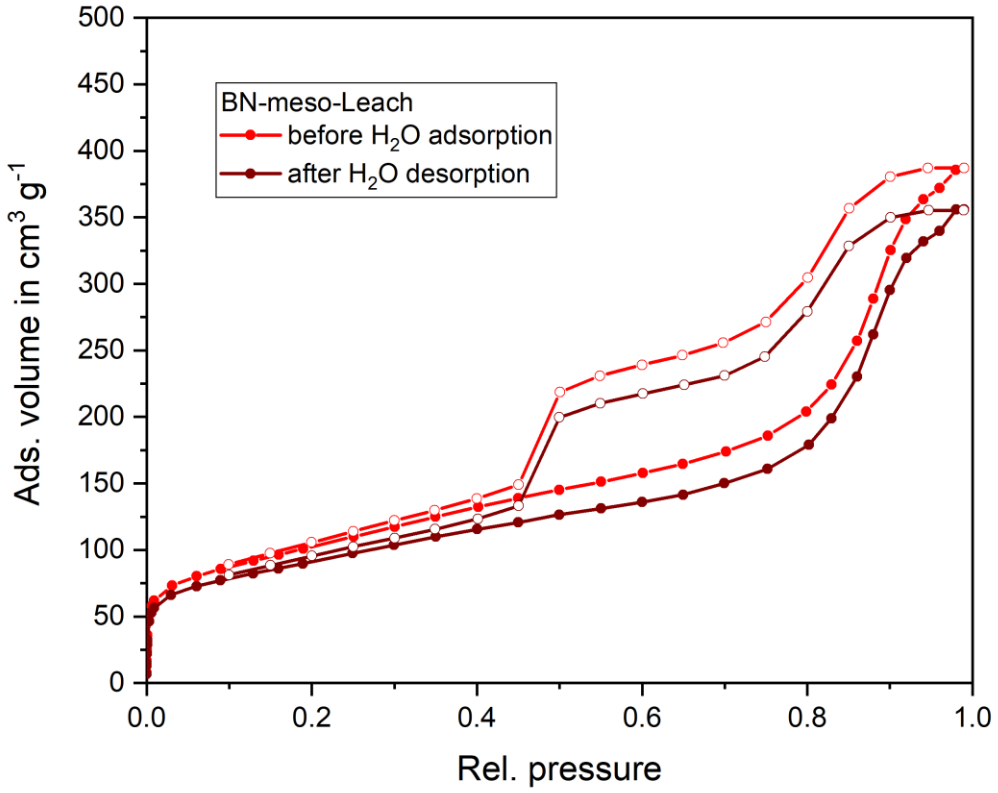

Figure S2: Nitrogen adsorption and desorption isotherms (77 K) before and after water adsorption and desorption.

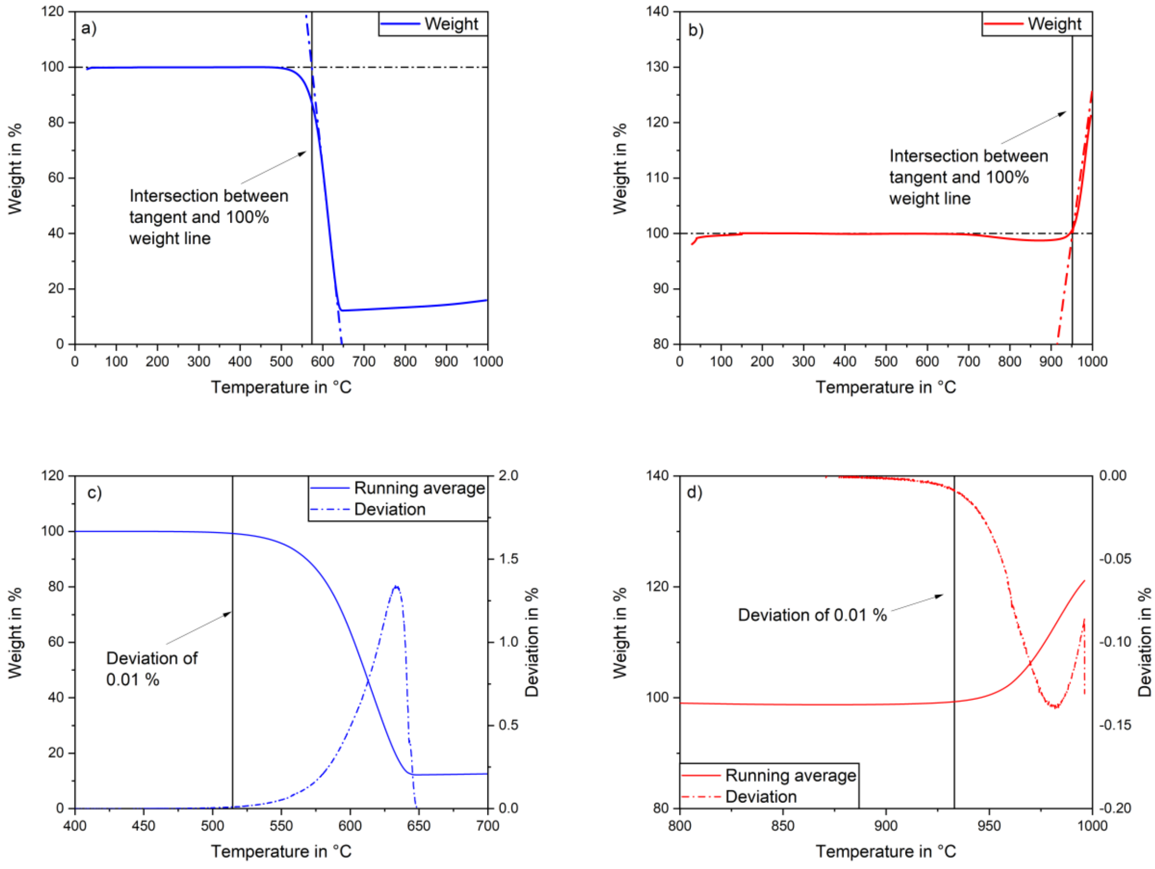

Figure S3: Graphical determination of SIT for a) C40 and b) BN-meso-Leach as well as of PIO for c) C40 and d) BN-meso-Leach.

Table S1: Freundlich parameters.

Adsorbent	Adsorptive	Freundlich parameter in mol kg^{-1} $mbar^{-n}$	Freundlich exponent	R^2
D47	Propane	0.8485	0.4598	0.9683
	Propanal	1.6894	0.3298	0.9983
	Acetone	2.1219	0.3136	0.9937
	Hexane	2.9766	0.1637	0.9711
	Toluene	3.9825	0.1821	0.9996
C40	Propane	0.2346	0.5990	0.9808
	Propanal	0.8540	0.4051	0.9960
	Acetone	1.1536	0.4063	0.9931
	Hexane	2.3602	0.2021	0.9939
	Toluene	3.6632	0.1108	0.9976
BN-meso	Propane	0.0256	0.4936	0.9731
	Propanal	0.0967	0.3064	0.9964
	Acetone	0.1158	0.2579	0.9943
	Hexane	0.1357	0.2767	0.9871
	Toluene	0.3762	0.2568	0.9998
BN-meso-Leach	Propane	0.0471	0.4147	0.9584
	Propanal	0.2356	0.3159	0.9947
	Acetone	0.2682	0.2832	0.9976
	Hexane	0.3189	0.2656	0.9826
	Toluene	0.7930	0.2538	0.9902

Table S2: Normalized freundlich parameters.

Adsorbent	Adsorptive	Freundlich parameter in µmol m_{micro}^{-2} $mbar^{-n}$	Freundlich exponent	R^2
D47	Propane	0.9283	0.4598	0.9683
	Propanal	1.8483	0.3298	0.9983
	Acetone	2.3216	0.3136	0.9937
	Hexane	3.2567	0.1637	0.9711
	Toluene	5.3100	0.1821	0.9996
C40	Propane	0.9155	0.5990	0.9808
	Propanal	1.1387	0.4051	0.9960
	Acetone	1.5382	0.4063	0.9931
	Hexane	3.1469	0.2021	0.9939
	Toluene	4.0079	0.1108	0.9976
BN-meso	Propane	0.4658	0.4936	0.9731
	Propanal	1.7574	0.3064	0.9964
	Acetone	2.1049	0.2579	0.9943
	Hexane	2.4677	0.2767	0.9871
	Toluene	6.8391	0.2568	0.9998
BN-meso-Leach	Propane	0.4456	0.4147	0.9584
	Propanal	2.2309	0.3159	0.9947
	Acetone	2.5399	0.2832	0.9976
	Hexane	3.0200	0.2656	0.9826
	Toluene	7.5100	0.2538	0.9902

Table S3: Acidic oxidic surface groups of D47 and C40.

Adsorbent	Surface group	Base consumption in µmol g^{-1}	Amount of substance equivalent in µmol g^{-1}
D47	Carboxyl	40.0	40.0
	Lactone / Lactol	131.1	91.1
	Phenol	178.3	47.2
	Carbonyl	314.3	136.0
C40	Carboxyl	21.0	21.0
	Lactone / Lactol	79.9	58.9
	Phenol	105.8	25.9
	Carbonyl	227.2	121.4

Table S4: Pore size distribution data of D47, C40, BN-meso and BN-meso-Leach.

Pore width in nm	D47 dV(log d) in cm³ g^{-1}	C40 dV(log d) in cm³ g^{-1}	BN-meso dV(log d) in cm³ g^{-1}	BN-meso-Leach dV(log d) in cm³ g^{-1}
0.567	0.5959	0	0	0
0.59	0.8681	0	0	0
0.614	1.3868	0	0	0
0.64	1.8346	0	0	0
0.666	2.0514	0.1492	0	0
0.694	1.9438	0.3202	0	0.0121
0.723	1.6493	0.7235	0	0.0559
0.753	1.2218	1.1659	0	0.1353
0.785	0.8132	1.5305	0	0.2348
0.818	0.5143	1.7524	0	0.3257
0.852	0.3437	1.7223	0	0.3890
0.889	0.3203	1.5366	0.0129	0.4111
0.926	0.4006	1.2381	0.0536	0.3910
0.966	0.5254	0.8955	0.1142	0.3363
1.007	0.6509	0.6074	0.1762	0.2628
1.051	0.7396	0.4206	0.2199	0.1852
1.096	0.7676	0.3495	0.2333	0.1202
1.144	0.7201	0.3761	0.2140	0.0735
1.193	0.6221	0.4656	0.1697	0.0463
1.245	0.5286	0.5741	0.1121	0.0327
1.299	0.4835	0.6760	0.0583	0.0265
1.356	0.4620	0.7484	0.0201	0.0241
1.416	0.4314	0.7998	0.0032	0.0254
1.478	0.3785	0.8295	0	0.0318
1.543	0.3186	0.8430	0	0.0406
1.611	0.2532	0.8606	0	0.0408
1.682	0.1559	0.8902	0	0.0172
1.756	0.0474	0.9106	0	0.0016
1.834	0	0.8764	0	0
1.915	0	0.7264	0	0
2.000	0	0.3196	0	0
2.121	0	0.0742	0	0
2.195	0	0.0138	0	0
2.272	0	0	0	0
2.351	0	0	0	0
2.434	0	0	0	0
2.519	0	0	0	0.0050
2.607	0	0.0279	0	0.0262
2.698	0	0.1500	0	0.0664
2.793	0	0.4076	0	0.1170
2.890	0	0.7752	0	0.1707
2.991	0	1.1765	0	0.2249

3.096	0	1.4972	0	0.2814
3.204	0	1.6488	0	0.3426
3.317	0	1.6247	0	0.4039
3.433	0	1.4721	0.0085	0.4519
3.553	0	1.2515	0.0371	0.4711
3.677	0	1.0174	0.0836	0.4525
3.806	0	0.8116	0.1354	0.3969
3.939	0	0.6528	0.1799	0.3121
4.077	0	0.5442	0.2065	0.2130
4.217	0	0.4769	0.2050	0.1120
4.367	0	0.4277	0.1687	0.0514
4.520	0	0.3738	0.1057	0.0173
4.678	0	0.3113	0.0425	0.0187
4.842	0	0.2473	0.0075	0.0458
5.012	0	0.1801	0	0.0844
5.187	0	0.1112	0	0.1233
5.369	0	0.0596	0	0.1523
5.556	0	0.0332	0	0.1709
5.751	0	0.0210	0	0.1855
5.952	0	0.0131	0.0109	0.1958
6.160	0	0.0075	0.0414	0.2017
6.376	0	0.0047	0.0793	0.2190
6.599	0	0.0031	0.1084	0.2620
6.830	0	0.0013	0.1194	0.3115
7.069	0	0.0002	0.1181	0.3449
7.317	0	0	0.1271	0.3806
7.573	0	0	0.1623	0.4572
7.838	0	0.0001	0.2041	0.5765
8.112	0	0.0014	0.2228	0.7055
8.396	0	0.0039	0.2245	0.8382
8.690	0	0.0058	0.2438	0.9906
8.994	0	0.0062	0.3047	1.1267
9.309	0	0.0059	0.3998	1.2043
9.635	0	0.0058	0.5221	1.2751
9.972	0	0.0054	0.6700	1.4027
10.321	0	0.0050	0.8012	1.5256
10.682	0	0.0047	0.8789	1.5660
11.056	0	0.0039	0.9520	1.5626
11.443	0	0.0035	1.0761	1.5594
11.844	0	0.0033	1.1970	1.5405
12.258	0	0.0032	1.2559	1.4682
12.687	0	0.0032	1.2977	1.3156
13.131	0	0.0032	1.3511	1.1045
13.591	0	0.0023	1.3838	0.9228
14.066	0	0.0027	1.3808	0.8222
14.559	0	0.0024	1.3553	0.7406
15.068	0	0.0023	1.3028	0.6055
15.596	0	0.0032	1.2358	0.4462
16.142	0	0.0067	1.1826	0.3392
16.707	0	0.0109	1.1178	0.3086
17.291	0	0.0126	0.9981	0.3163
17.896	0	0.0119	0.8441	0.3053
18.523	0	0.0114	0.7258	0.2669
19.171	0	0.0118	0.6874	0.2160
19.842	0	0.0111	0.6892	0.1638
20.537	0	0.0099	0.6443	0.1327
21.255	0	0.0096	0.5472	0.1317
21.999	0	0.0094	0.4388	0.1399
22.769	0	0.0084	0.3425	0.1359
23.566	0	0.0073	0.2905	0.1202
24.391	0	0.0078	0.2938	0.1038
25.245	0	0.0081	0.3125	0.1016
26.128	0	0.0072	0.3088	0.1287
27.043	0	0.0068	0.2838	0.1842
27.989	0	0.0067	0.2447	0.2224

28.969	0	0.0063	0.1933	0.2276
29.983	0	0.0075	0.1567	0
31.032	0	0.0120	0.1657	0
32.118	0	0.0152	0.1892	0
32.242			0.1949	0

Table S5: Water-vapor isotherm data of D47, C40, BN-meso and BN-meso-Leach at 25°C.

D47		C40		BN-meso		BN-meso-Leach	
Relative humidity	Adsorbed volume in cm^3 g^{-1}	Relative humidity	Adsorbed volume in cm^3 g^{-1}	Relative humidity	Adsorbed volume in cm^3 g^{-1}	Relative humidity	Adsorbed volume in cm^3 g^{-1}
ADS		ADS		ADS		ADS	
0.01315	0.7184	0.0133	0.5925	0.0106	9.0296	0.01171	4.4894
0.05184	1.8194	0.0520	1.6313	0.0498	21.1849	0.05302	10.2011
0.09788	2.7911	0.1019	2.6221	0.0970	29.1171	0.09754	14.0806
0.14773	3.8922	0.1513	3.6078	0.1472	33.4563	0.15251	18.2768
0.20084	5.3385	0.1973	4.6284	0.1971	37.5090	0.19919	21.6376
0.25089	7.1553	0.2514	6.2337	0.2473	42.3957	0.24831	25.4370
0.29898	9.9466	0.3011	8.4203	0.2973	45.9544	0.30021	30.0319
0.34931	14.5891	0.3501	11.8409	0.3472	50.0174	0.34987	34.7385
0.39872	21.9600	0.4003	17.2150	0.3989	55.7378	0.39791	40.4985
0.45049	32.3727	0.4479	25.5011	0.4491	61.8503	0.44857	46.8909
0.49839	46.1929	0.4984	43.6566	0.5018	69.9789	0.49969	54.8510
0.54777	75.9061	0.5481	81.9183	0.5472	78.6268	0.54877	64.4321
0.59824	117.3549	0.5983	129.6232	0.5980	93.6063	0.59823	76.5892
0.64782	157.9190	0.6475	177.9277	0.6479	110.8885	0.64824	92.5632
0.69776	204.2909	0.6978	231.9715	0.6975	130.8428	0.69806	115.1687
0.74715	252.2893	0.7482	290.7461	0.7479	152.4366	0.74841	143.4229
0.79935	299.9826	0.7986	349.6290	0.7982	175.3985	0.79767	172.2298
0.85009	347.8705	0.8498	406.0035	0.8482	195.4424	0.84736	200.1020
0.89807	388.0371	0.9004	445.8147	0.8983	222.7552	0.89824	239.2089
0.96246	392.5400	0.9500	448.4654				
0.98921	395.4310	0.9850	451.4864				
DES		DES		DES		DES	
0.9892	395.4310	0.9850	451.4864	0.8952	222.7087	0.8943	239.0596
0.9425	393.6540	0.9615	451.2367	0.8491	215.2061	0.8490	228.3380
0.8981	389.8371	0.9004	450.2367	0.7985	206.9579	0.8022	217.3780
0.8942	388.0073	0.8962	448.7355	0.7504	199.5356	0.7493	205.2008
0.8485	382.9018	0.8528	443.2524	0.7027	192.1378	0.6992	193.2107
0.7959	375.2359	0.7965	434.4830	0.6507	183.9164	0.6503	180.3778
0.7507	364.6356	0.7491	423.6818	0.6026	175.0106	0.6001	162.2033
0.7009	347.0834	0.6996	407.0096	0.5530	165.0387	0.5525	131.6618
0.6496	321.7894	0.6518	380.5706	0.5028	150.5158	0.5015	94.8604
0.6016	284.7548	0.6027	335.0746	0.4527	132.9076	0.4498	77.2905
0.5527	200.8583	0.5531	222.5733	0.4028	116.9970	0.4018	67.4424
0.5026	79.6591	0.5026	81.0656	0.3528	103.7826	0.3499	58.5509
0.4490	47.6644	0.4454	38.7436	0.3017	93.9732	0.3001	51.5109
0.3967	34.2583	0.4000	28.5074	0.2516	85.6886	0.2506	45.5696
0.3510	26.8126	0.3529	22.1770	0.2013	78.3972	0.1997	40.1960
0.3024	21.2054	0.3023	17.8398	0.1518	72.2693	0.1503	35.3425
				0.1024	67.2154	0.0997	31.0439
				0.0527	62.1883	0.0523	26.8630
				0.0109	54.0810	0.0105	17.8463

Table S6: Adsorption isotherm data of toluene, n-hexane, acetone, propanal, and propane on D47 and C40 at 25°C.

D47			C40		
Partial pressure in mbar	Loading in mol kg^{-1}	Loading in µmol m$^{-2}_{micro}$	Partial pressure in mbar	Loading in mol kg^{-1}	Loading in µmol m$^{-2}_{micro}$
toluene					
0.04	2.5382	2.9460	0.04	2.2095	2.7770
0.13	2.9430	3.6903	0.13	2.7677	3.2199
0.23	3.1262	4.0686	0.23	3.0515	3.4204
0.33	3.2432	4.3215	0.33	3.2411	3.5484
0.43	3.3575	4.5384	0.43	3.4038	3.6735
0.53	3.3953	4.7216	0.53	3.5412	3.7147
0.63	3.4872	4.8787	0.63	3.6590	3.8154
0.73	3.5360	5.0154	0.73	3.7616	3.8687
0.83	3.5743	5.1358	0.83	3.8518	3.9106
0.93	3.6284	5.2550	0.93	3.9412	3.9698
n-hexane					
0.04	1.6511	1.8064	0.04	1.1820	1.5761
0.13	2.1498	2.3520	0.13	1.5846	2.1128
0.23	2.3691	2.5920	0.23	1.7642	2.3523
0.33	2.5946	2.8387	0.33	1.9080	2.5440
0.43	2.6592	2.9095	0.43	2.0132	2.6843
0.53	2.7138	2.9692	0.53	2.0839	2.7786
0.63	2.7464	3.0048	0.63	2.1682	2.8909
0.73	2.7931	3.0559	0.73	2.2176	2.9568
0.83	2.8513	3.1195	0.83	2.2593	3.0124
0.93	2.8684	3.1383	0.93	2.2794	3.0391
acetone					
0.02	0.57439	0.62844	0.03	0.25647	0.34196
0.11	1.05305	1.15214	0.12	0.48399	0.64532
0.21	1.31679	1.44069	0.22	0.62863	0.83818
0.31	1.48145	1.62084	0.32	0.72808	0.97078
0.41	1.66932	1.82638	0.42	0.85171	1.13562
0.51	1.70956	1.87042	0.52	0.89487	1.19316
0.61	1.81499	1.98577	0.62	0.94553	1.26070
0.71	1.92455	2.10564	0.72	0.9653	1.28707
0.81	1.99517	2.18290	0.82	1.05543	1.40725
0.91	1.99295	2.18047	0.92	1.13222	1.50963
propanal					
0.04	0.57901	0.63349	0.04	0.22738	0.30318
0.13	0.86091	0.94191	0.13	0.37955	0.50606
0.23	1.03075	1.12773	0.23	0.47565	0.63420
0.33	1.17208	1.28237	0.33	0.53807	0.71743
0.43	1.28307	1.40379	0.43	0.59382	0.79176
0.53	1.40787	1.54034	0.53	0.68613	0.91484
0.63	1.44179	1.57745	0.63	0.69728	0.92971
0.73	1.52084	1.66394	0.73	0.74142	0.98856
0.83	1.57753	1.72596	0.83	0.80395	1.07193
0.93	1.64354	1.79818	0.93	0.82598	1.10131
propane					
0.04	0.14084	0.15409	0.04	0.02488	0.09710
0.13	0.32746	0.35827	0.13	0.06507	0.25392
0.23	0.46128	0.50468	0.23	0.09796	0.38230
0.33	0.52640	0.57593	0.33	0.12331	0.48120
0.43	0.59321	0.64902	0.43	0.15393	0.60071
0.53	0.65885	0.72085	0.53	0.16319	0.63686
0.73	0.68552	0.75002	0.73	0.18405	0.71823

Table S7: Adsorption isotherm data of toluene, n-hexane, acetone, propanal, and propane on BN-meso and BN-meso-Leach at 25°C.

BN-meso			BN-meso-Leach		
Partial pressure in mbar	Loading in mol kg^{-1}	Loading in µmol m$^{-2}_{micro}$	Partial pressure in mbar	Loading in mol kg^{-1}	Loading in µmol m$^{-2}_{micro}$
toluene					
0.04	0.16564	3.01169	0.04	0.35395	3.35214
0.13	0.22268	4.04880	0.13	0.48398	4.58355
0.23	0.25894	4.70800	0.23	0.55046	5.21321
0.33	0.28242	5.13497	0.33	0.59605	5.64498
0.43	0.30151	5.48194	0.43	0.61937	5.86582
0.53	0.31535	5.73361	0.53	0.65084	6.16380
0.63	0.33594	6.10794	0.63	0.70122	6.64100
0.73	0.34608	6.29230	0.73	0.75033	7.10605
0.83	0.36288	6.59776	0.83	0.7643	7.23839
0.93	0.36818	6.69415	0.93	0.78537	7.43792
n-hexane					
0.04	0.05153	0.93695	0.04	0.12192	1.15461
0.13	0.07597	1.38134	0.13	0.18598	1.76135
0.23	0.09170	1.66721	0.23	0.22390	2.12050
0.33	0.10253	1.86416	0.33	0.23992	2.27216
0.43	0.11122	2.02211	0.43	0.26194	2.48070
0.53	0.11602	2.10946	0.53	0.27715	2.62474
0.63	0.11986	2.17926	0.63	0.28635	2.71191
0.73	0.12520	2.27641	0.73	0.28994	2.74588
0.83	0.12722	2.31302	0.83	0.29839	2.82594
0.93	0.12819	2.33064	0.93	0.30281	2.86775
acetone					
0.04	0.05132	0.93304	0.04	0.10758	1.01889
0.13	0.06523	1.18599	0.13	0.14395	1.36327
0.23	0.08112	1.47491	0.23	0.17831	1.68870
0.33	0.08837	1.60669	0.33	0.19815	1.87659
0.43	0.09279	1.68702	0.43	0.21270	2.01442
0.53	0.09704	1.76438	0.53	0.22875	2.16637
0.63	0.10384	1.88807	0.63	0.23833	2.25714
0.73	0.10698	1.94503	0.73	0.24828	2.35140
0.83	0.11091	2.01663	0.83	0.25161	2.38286
0.93	0.11240	2.04366	0.93	0.25598	2.42431
propanal					
0.04	0.03710	0.67453	0.04	0.08301	0.78616
0.13	0.05205	0.94628	0.13	0.12419	1.17611
0.23	0.06193	1.12591	0.23	0.14808	1.40238
0.33	0.06835	1.24280	0.33	0.16258	1.53973
0.43	0.07291	1.32561	0.43	0.18506	1.75267
0.53	0.07810	1.41999	0.53	0.19300	1.82782
0.63	0.08319	1.51263	0.63	0.20527	1.94401
0.73	0.08904	1.61894	0.73	0.21827	2.06712
0.83	0.09288	1.68878	0.83	0.22120	2.09490
0.93	0.09461	1.72016	0.93	0.22407	2.12207
propane					
0.04	0.00353	0.06423	0.04	0.00907	0.08590
0.13	0.00945	0.17187	0.13	0.02019	0.19119
0.23	0.01284	0.23344	0.23	0.02685	0.25431
0.33	0.01578	0.28692	0.33	0.03221	0.30507
0.43	0.01770	0.32187	0.43	0.03460	0.32765
0.53	0.01864	0.33895	0.53	0.03590	0.34004
0.73	0.02085	0.37908	0.73	0.03876	0.36705

Table S8: Adsorption isotherm and heat of adsorption data of acetone, toluene, and n-hexane on D47 and BN-meso at 25°C.

D47			BN-meso		
Relative pressure	Loading in mol kg^{-1}	Heat of adsorption in kJ mol^{-1}	Relative pressure	Loading in mol kg^{-1}	Heat of adsorption in kJ mol^{-1}
Acetone					
0.00005	0.19579	55.66062	0.00057	0.06944	57.38434
0.00027	0.57977	53.25968	0.00326	0.13663	46.82155
0.00069	0.96774	50.43124	0.01690	0.27147	40.19126
0.00147	1.35282	48.58582	0.06356	0.59177	39.02540
0.00202	1.55514	48.53200	0.10039	0.80133	37.45873
0.00355	1.95690	47.79639	0.16989	1.11426	37.21155
0.00575	2.35607	46.43882	0.21957	1.28812	35.02640
0.00894	2.75074	46.49282	0.30514	1.50375	36.97881
0.01102	2.94575	44.77998	0.41161	1.70068	37.38585
0.01517	3.24159	45.42776	0.51164	1.88775	35.07560
0.02085	3.53270	45.35172	0.62010	2.15004	33.72680
0.02874	3.81449	45.41051	0.70846	2.52088	33.29792
0.03932	4.08187	44.69421	0.77931	3.19394	34.12444
0.05499	4.33216	45.90386	0.80328	3.61198	33.27733
0.07749	4.55430	45.27527	0.83033	4.35353	34.07140
0.10855	4.74436	46.15377	0.85256	4.86826	34.15890
0.17912	4.95851	46.9706	0.87466	5.46827	33.45581
0.25987	5.08209	48.82189	0.90413	5.61742	33.38347
0.37089	5.18917	49.54305	0.96102	5.75146	32.51178
0.40000	5.27304	49.96256			
0.60564	5.34041	45.52303			
0.75964	5.42563	47.1527			
0.86849	5.49880	37.37302			
0.93584	5.57553	30.43936			
0.96613	5.67146	23.19446			
toluene					
0.00012	0.06690	62.01251	0.00031	0.03327	58.88732
0.00063	0.26759	63.44280	0.00065	0.06650	61.57058
0.00115	0.46868	68.68300	0.00113	0.09965	52.73344
0.00178	0.73566	63.74297	0.00915	0.30491	59.91900
0.00242	1.00205	65.62667	0.01464	0.37270	53.10795
0.00309	1.26791	64.34779	0.06416	0.63380	50.56734
0.00348	1.53368	62.70642	0.10940	0.75792	48.84879
0.00427	1.79847	65.04406	0.21468	0.92933	45.89332
0.00525	2.06246	60.47741	0.32503	1.08472	42.59421
0.00665	2.32480	61.13758	0.42013	1.22490	46.67000
0.00883	2.58655	63.17092	0.50656	1.39712	43.79797
0.01446	2.84461	64.44817	0.60489	1.70652	40.65607
0.02520	3.09762	61.31332	0.69352	2.31574	43.31178
0.05480	3.34438	60.51232	0.70000	2.38065	41.27822
0.10666	3.45891	63.67297	0.75077	2.99817	42.99184
0.22112	3.55903	63.19703	0.79616	3.61943	42.19118
0.39852	3.63835	63.64816	0.80265	3.69814	45.73176
0.50225	3.66936	61.94585	0.86640	4.32100	41.69757
0.67947	3.72053	56.16149	0.90963	4.50656	51.49941
0.75185	3.74190	47.99837	0.94960	4.61899	28.69039
0.81452	3.76095	52.76237	0.98095	4.72428	29.76420
0.90423	3.79468	42.15556			
0.95608	3.82587	41.35871			
0.98743	3.85670	47.09434			

n-hexane					
0.00057	0.18579	68.78897	0.00022	0.02788	62.66721
0.00122	0.55787	64.46675	0.00123	0.10347	56.14783
0.00155	0.94303	60.52371	0.01025	0.34263	47.78910
0.00185	1.32685	64.01500	0.06226	0.59481	43.01624
0.00232	1.70817	58.46376	0.11139	0.68355	37.87839
0.00341	2.08655	60.56681	0.25227	0.83979	37.79107
0.00895	2.45815	58.05663	0.33205	0.91916	29.51550
0.01914	2.63608	55.47326	0.41711	1.01316	34.15402
0.19726	2.95989	60.51864	0.54175	1.19327	29.73944
0.37184	3.02298	55.23326	0.62192	1.38195	32.59319
0.58255	3.07263	50.53065	0.71635	1.85536	32.83837
0.77540	3.11747	36.64786	0.77624	2.50406	33.28599
0.90268	3.15878	31.94438	0.80994	2.95320	33.20605
0.96441	3.21281	30.20096	0.89374	3.64995	33.00835
0.98976	3.28589	19.55396	0.92604	3.74302	32.97111
			0.95770	3.82694	32.77750
			0.98509	4.10855	22.91654
